# Historically unprecedented Northern Gulf of Mexico hurricane activity from 650 to 1250 CE

**DOI:** 10.1038/s41598-020-75874-0

**Published:** 2020-11-05

**Authors:** Jessica R. Rodysill, Jeffrey P. Donnelly, Richard Sullivan, Philip D. Lane, Michael Toomey, Jonathan D. Woodruff, Andrea D. Hawkes, Dana MacDonald, Nicole d’Entremont, Kelly McKeon, Elizabeth Wallace, Peter J. van Hengstum

**Affiliations:** 1 Florence Bascom Geoscience Center, United States Geological Survey, Reston, VA USA; 2 Geology and Geophysics, Woods Hole Oceanographic Institution, Woods Hole, MA USA; 3 Department of Earth, Environmental and Planetary Sciences, Brown University, Providence, RI USA; 4 Department of Oceanography, Texas A&M University, College Station, TX USA; 5 Department of Geosciences, University of Massachusetts, Amherst, MA USA; 6 Department of Earth and Ocean Sciences, University of North Carolina Wilmington, Wilmington, NC USA; 7 Department of Marine Sciences, Texas A&M University at Galveston, Galveston, TX USA

**Keywords:** Climate sciences, Limnology, Natural hazards, Ocean sciences

## Abstract

Hurricane Michael (2018) was the first Category 5 storm on record to make landfall on the Florida panhandle since at least 1851 CE (Common Era), and it resulted in the loss of 59 lives and $25 billion in damages across the southeastern U.S. This event placed a spotlight on recent intense (exceeding Category 4 or 5 on the Saffir-Simpson Hurricane Wind Scale) hurricane landfalls, prompting questions about the natural range in variability of hurricane activity that the instrumental record is too short to address. Of particular interest is determining whether the frequency of recent intense hurricane landfalls in the northern Gulf of Mexico (GOM) is within or outside the natural range of intense hurricane activity prior to 1851 CE. In this study, we identify intense hurricane landfalls in northwest Florida during the past 2000 years based on coarse anomaly event detection from two coastal lacustrine sediment archives. We identified a historically unprecedented period of heightened storm activity common to four Florida panhandle localities from 650 to 1250 CE and a shift to a relatively quiescent storm climate in the GOM spanning the past six centuries. Our study provides long-term context for events like Hurricane Michael and suggests that the observational period 1851 CE to present may underrepresent the natural range in landfalling hurricane activity.

## Introduction

Tropical cyclones are a serious threat for densely populated coastal communities, particularly the Florida panhandle^1^, where growing concentrations of people and properties have resulted in a steady increase in damage from hurricane landfalls^2^. The frequency and intensity of tropical cyclones have varied substantially over the past several decades^3–7^ and are thought to be largely controlled by sea surface temperature (SST) variations, wind shear, and upper tropospheric temperatures^8–12^. Warmer SSTs in the tropical Atlantic main development region (MDR), cooler upper troposphere temperatures, and reduced wind shear correspond to a higher number of intense hurricanes in the Atlantic basin^8–13^.

Additional mechanisms are thought to influence tropical cyclone activity in the Gulf of Mexico (GOM) region, including the Loop Current^14,15^, the El Niño-Southern Oscillation^11^, and factors controlling cyclogenesis (e.g. SSTs, wind shear, and upper troposphere temperatures) in the deep tropics on the western half of the Atlantic Ocean and within the GOM, where the majority of historical Gulf Coast landfalling hurricanes have formed^16^. The Loop Current extends into the northern GOM during boreal summer when the Intertropical Convergence Zone (ITCZ) seasonally migrates northward, deepening the thermocline during summer and early fall^17^. A shallow thermocline allows for deeper, colder water to be incorporated into the mixed layer during high energy storm conditions, reducing the vertical temperature gradient and weakening the tropical cyclone^18^. A deeper, warmer surface layer in the GOM produces more favorable conditions for maintaining tropical cyclone strength. Further, a reduction in upper atmospheric wind shear during La Niña years tends to correspond to more frequent hurricane activity in the Atlantic basin through atmospheric teleconnections^19,20^. Within the GOM, hurricanes can also be enhanced when the thermal gradient between the surface water and the atmosphere is strengthened due to a cooling of the lower stratosphere, as occurs during El Niño events^11^. The manner in which SSTs, winds, and atmospheric temperatures collectively control storm patterns on centennial and longer timescales is less certain, and how future changes in the mean climate state and radiative forcing will influence hurricane climatology is unclear.

Theory, modeling, and analyses of the short historical hurricane record have led to contradicting hypotheses on whether Atlantic tropical cyclone frequency and intensity will increase, decrease, or remain unchanged in the near future^3,4,21–25^. Recent improvements in hurricane simulation through increased general circulation model horizontal resolution^26^ and downscaling (e.g.^27^) has led to several model-based predictions that the proportion of high intensity hurricanes in the North Atlantic will increase in response to increased radiative forcing (e.g.^4,5,23–25,28–32^). Colbert et al.^33^ predict an eastward shift in cyclogenesis in the tropical Atlantic Ocean and a westerly wind anomaly in the southern GOM and Caribbean Sea that will lead to a reduction in the number of storm tracks leading into the GOM over the coming century. Conversely, intense tropical cyclone activity increased in the GOM over the coming century under representative concentration pathway 4.5 (RCP 4.5), simulated using the Geophysical Fluid Dynamics (GFDL) High Resolution Atmospheric Model (HiRAM) downscaled into the GFDL hurricane model, while global frequency of all tropical cyclones decreased^24,27^. The relative importance of the various mechanisms controlling tropical cyclone variability within the GOM is unknown, and the baseline range of hurricane activity for the GOM is not well-established prior to the year 1851 CE.

Previously published tropical cyclone reconstructions from the northeastern GOM leave an incomplete picture of prehistoric trends in regional hurricane activity over the Common Era (e.g.^14,34–36^). Liu and Fearn^34,35^ suggest there were frequent intense storms until 1250 CE at Lake Shelby in Alabama and until 950 CE at Western Lake in northwest Florida, based on the visual identification of overwash deposits in lake sediment cores, but evidence for only one strong storm was observed after 1250 CE. Reconstructions of intense hurricane overwash deposits based on grain size variations in sediment cores from Mullet Pond and Spring Creek Pond in northwest Florida identify an active interval from 450 to 1350 CE with quiescent periods from 50 to 350 CE and from 1550 CE to present^14,36^. Whether the differences between these reconstructions are caused by spatial variations in landfalling hurricanes or site-specific factors, such as chronological control, storm identification methods, or site sensitivity, remains uncertain. In this study, we attempt to elucidate regional tropical cyclone variability along the northeastern GOM coast during the Common Era by reconstructing intense hurricane activity in two locations proximal to existing reconstructions (Western Lake and Mullet Pond): Basin Bayou and Shotgun Pond.

**Figure 1:**
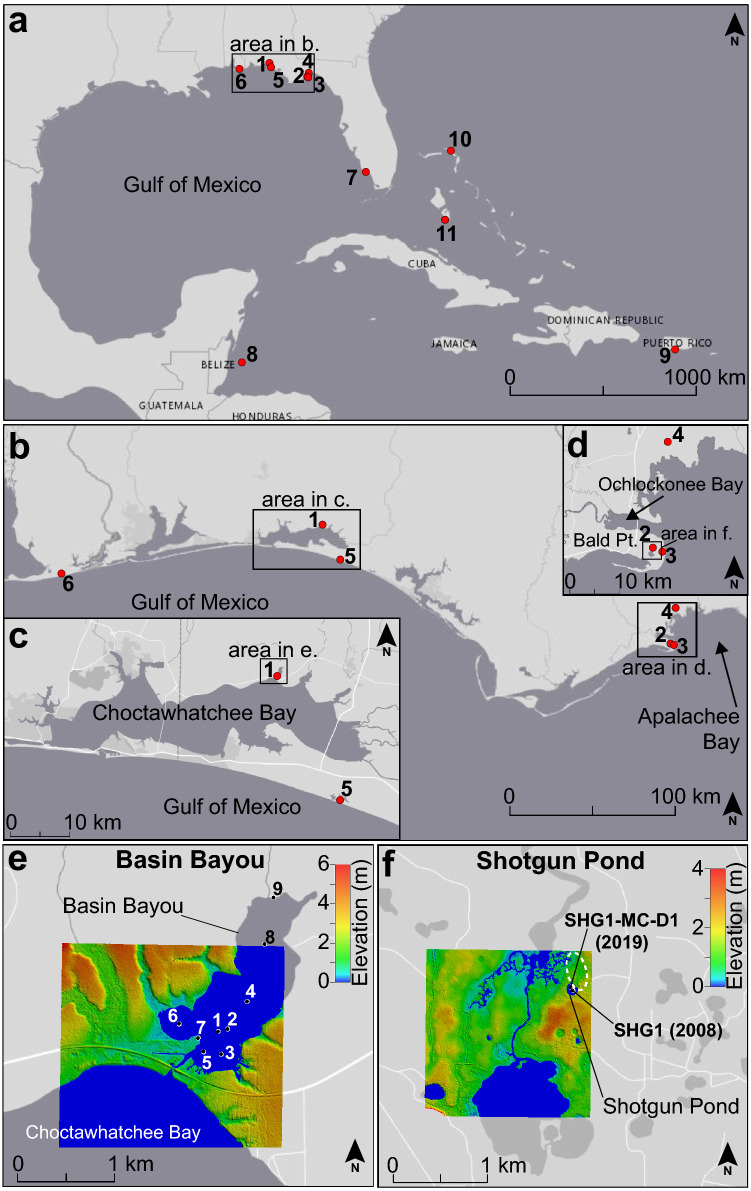
Location maps. (**a)** Gulf of Mexico and Caribbean region with red circles indicating the locations of sites discussed in text, including: 1: Basin Bayou (this study), 2: Shotgun Pond (this study), 3: Mullet Pond^36^, 4: Spring Creek Pond^14^, 5: Western Lake^35^, 6: Lake Shelby^34^, 7: Island Bay^45^. 8: Lighthouse Reef, Belize^46^, 9: Laguna Playa Grande, Vieques, Puerto Rico^49^, 10: Blackwood Sinkhole, The Bahamas^48^, and 11: South Andros Island, The Bahamas^47^. (**b**) Locations of tropical cyclone reconstructions on the northeastern Gulf of Mexico coast indicated by red circles. Site numbers are the same as in panel **a**. (**c**) Map of Choctawhatchee Bay and the locations of Basin Bayou and Western Lake (red circles). Site numbers are the same as in panel **a**. (**d)** Map of the Bald Point peninsula in Apalachee Bay and the locations of Shotgun Pond, Mullet Pond, and Spring Creek Pond (red circles). Site numbers are the same as in panel **a**. (**e**) Core locations (black dots) and Lidar elevation map of Basin Bayou. Elevation is in meters with warmer colors indicating higher elevations. Lidar data was collected in 2006 at 1 cm per pixel resolution with an elevation uncertainty of 13 cm^63^. (**f**) Core locations (black dots) and Lidar elevation map of Shotgun Pond. Elevation is in meters with warmer colors indicating higher elevations. Lidar data was collected in 2006 at 1 cm per pixel resolution with an elevation uncertainty of 13 cm^63^. The lowest elevation floodwater route on the north end of the pond discussed in the text is outlined with a white dashed oval. Maps were generated using ArcMap v. 10.6 (https://desktop.arcgis.com/en/arcmap/). Basemaps were provided by the Esri, HERE, Garmin, OpenStreetMap contributors and the GIS user community. Lidar maps were generated using Global Mapper v. 21.0 (www.globalmapper.com).

Basin Bayou (30.4897ºN, 86.2463ºW) is located on the northeast side of Choctawhatchee Bay in northwest Florida, 21 km northwest of Western Lake (Fig. 1). The bayou is 1.5 m deep and surrounded by unconsolidated Pleistocene and Holocene-aged siliciclastic sand and clays^37^. A small stream, Basin Creek, drains into the north end of Basin Bayou from a relatively small watershed (117.5 km^2^), representing 2.5% of the Choctawhatchee Bay catchment area^38^. On the south end of the bayou, a 250–400 m-wide baymouth barrier separates Basin Bayou from Choctawhatchee Bay. A narrow channel cuts through the barrier and is a conduit for tidal water flow between the bayou and the bay; tidal range within Choctawhatchee Bay is minimal, averaging 0.15 m^39^. Based on light detection and ranging (Lidar) elevation data, the transition between the collision regime, where wave runup is confined to the bay side of the barrier, and the overwash regime, where waves overtop the barrier^40^, is at 1.1 + /- 0.13 m above sea level (masl). Complete inundation of the baymouth barrier, the inundation regime^40^, is achieved for storm tides exceeding 1.8 masl, at present. We investigate the potential for overwash and inundation regime flooding during historical storms below. The sensitivity of Basin Bayou to overwash and inundation relies on the stability of these regime elevations over time, which are influenced by dynamic coastal processes (e.g. barrier evolution) and sea level changes. Distinguishing the relative importance of diminishing site-to-sea distance and barrier evolution on the susceptibility of Basin Bayou to storm deposition is challenging. For this reason, we focus this study on the past 2000 years, a time of relatively stable sea level and uniform background sediment deposition (Supplementary Information).

Shotgun Pond (29.9316ºN, 84.355ºW) is a sinkhole pond on the Bald Point peninsula, which is west of Apalachee Bay, approximately 200 km southeast of Basin Bayou, and 1.7 km west of Mullet Pond (Fig. 1). The freshwater pond is 5 m deep and about 70 m wide with no tidal influence^15^. Similar to Basin Bayou, the land surface surrounding Shotgun Pond is predominantly Holocene and Pleistocene aged fine quartz sand that is underlain by limestone and dolomite bedrock^37^. Lidar elevation data indicates that much of the eastern half of the Bald Point peninsula lies below 2 masl, with dunes and relic dune features reaching up to 4 masl to the southeast and 15 m to the west of Shotgun Pond. The lowest elevation connection between Shotgun Pond and open water is through a tidal marsh and channel system extending from Ochlockonee Bay to within 80 m of the north of the pond; storm-induced flooding and subsequent sand deposition could occur via this route with a minimum storm tide of just over a meter^41^. For Shotgun Pond, the transition between the collision and overwash regimes is 1.1 + /- 0.13 masl, and the inundation regime is reached when storm tides exceed 5 masl^41^. Similar to Basin Bayou, we focus on a time of relatively stable sea level and uniform background sedimentation in Shotgun Pond in an effort to minimize the impacts of changing site sensitivity through time.

## Results and discussion

### Historical tropical cyclone-driven flooding

At both Basin Bayou and Shotgun Pond, storm surges were simulated using the Sea, Lake, and Overland Surges from Hurricanes (SLOSH) model across the range of observed historical storm parameters in the Extended Best-Track data set^6^. We corrected the modeled storm surges for local tide conditions using tide gauge measurements and integrated these storm tide estimates with Lidar elevation data to identify the ranges of historical storm intensities and proximities that were most likely to inundate each site. Elevation data indicates that a minimum local storm tide exceeding ~ 1.1 m is necessary for inundation to reach Basin Bayou and Shotgun Pond in their modern configurations, providing a minimum storm tide elevation constraint on storm-induced flooding. Extensive, thick sheets of sand are most likely deposited when local storm tides reach the inundation regime, such that the barriers at each site are subjected to surf-zone processes^40^.

**Figure 2:**
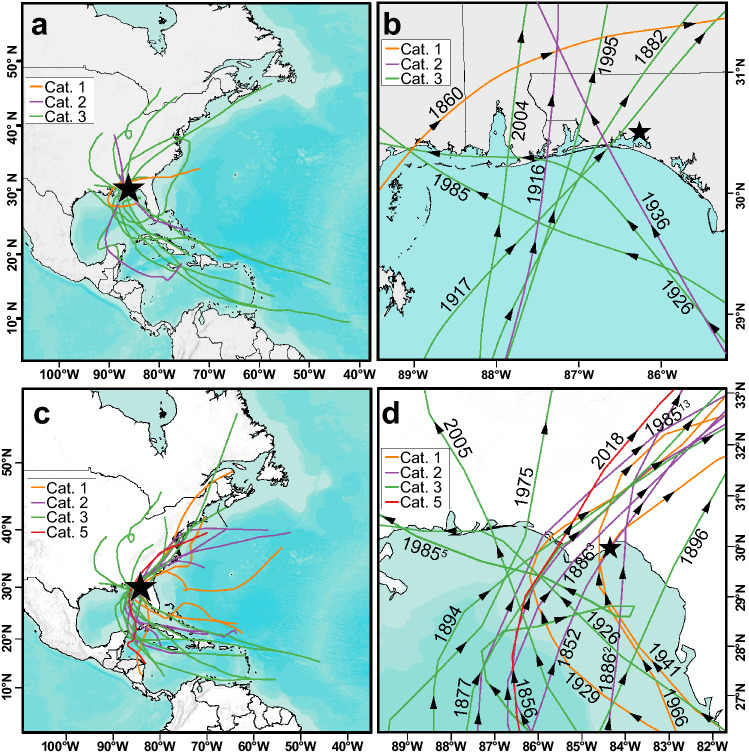
Storm tracks of historical tropical cyclones. (**a**) The location of Basin Bayou is indicated by a black star. Storm tracks for the Category 1 (orange), Category 2 (purple), and Category 3 (green) tropical cyclones that produced overwash and inundation regime modeled surges (> 1.1 m) at Basin Bayou are shown. (**b**) Close-up of panel **a**. The years label the storm season for each landfall, and black arrows indicate the direction of each hurricane along its track. Named storms shown are Elena (1985), Opal (1995), and Ivan (2004). (**c**) Storm tracks for the Category 1 (orange), Category 2 (purple), Category 3 (green), and Category 5 (red) tropical cyclones that produced overwash and inundation regime modeled surges (> 1.1 m) at Shotgun Pond are shown. Shotgun Pond is indicated by a black star. (**d**) The same as in panel **b** for Shotgun Pond. Superscripts indicate the Best-Track storm number for seasons with more than one tropical cyclone displayed within the map area. Named storms shown are Alma (1966), Eloise (1975), Elena (1985, storm number 5), Kate (1985, storm number 13), Dennis (2005), and Michael (2018). Maps were generated using ArcMap v. 10.6 (https://desktop.arcgis.com/en/arcmap/). Basemaps were provided by the Esri, HERE, Garmin, OpenStreetMap contributors and the GIS user community.

**Figure 3:**
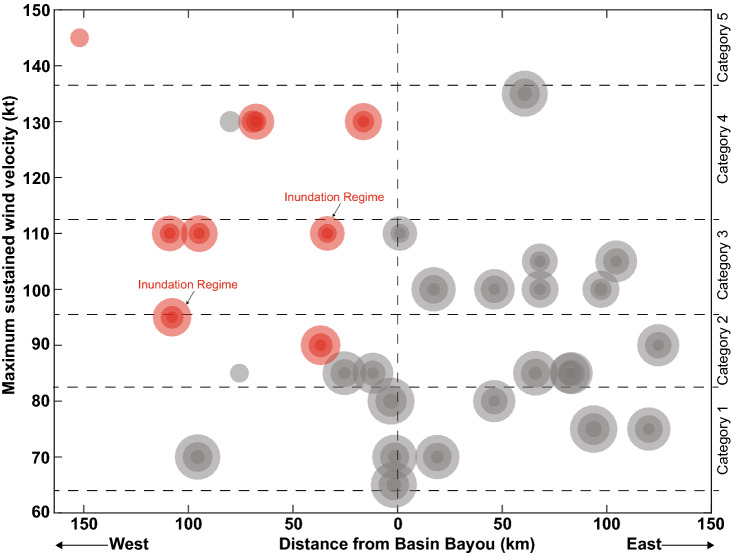
Historical tropical cyclones passing within 150 km of Basin Bayou are plotted by the distance of landfall from Basin Bayou in km (x axis) and the maximum sustained wind velocity in knots (y axis) *during the lifetime of the storm* (i.e. not the max. sustained wind velocity at the time of landfall). The diameters of the circles represent the estimated or observed radii of the storms, shading denotes the median modelled radius of maximum winds + / − 1 sigma, and the sizes of the circles scale from the minimum radius (16 km) to the maximum radius (79 km). The red circles represent storms that produced modeled surges exceeding the minimum flood threshold (1.1 m) at Basin Bayou, and the gray circles represent storms that did not produce modeled surges exceeding 1.1 m. Storm tides exceeded the inundation regime threshold (1.8 m) for two modeled hurricanes, labeled “Inundation Regime.”

**Figure 4:**
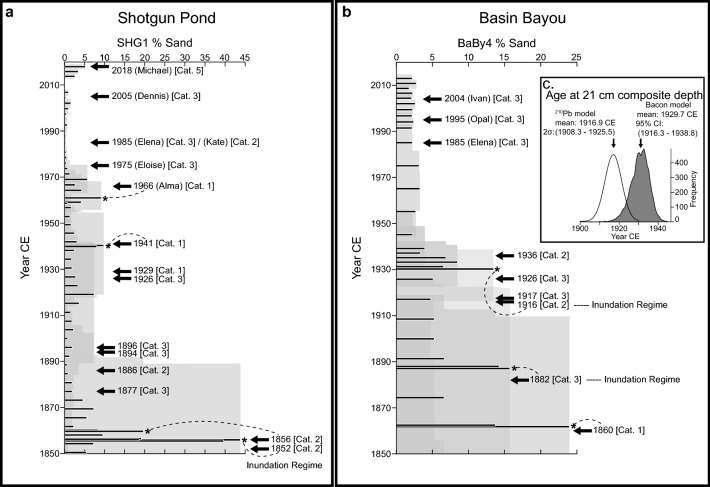
Sand contents from 1850 CE to present in Shotgun Pond (**a**) and Basin Bayou (**b**) are plotted alongside historical hurricanes with modeled storm tides (SLOSH-modeled surge plus tide) that reached the overwash and inundation regimes at each site. Percent sand values identified as “events” are marked with an asterisk. The gray shading represents the age uncertainty for each time series. Dashed lines connect event deposits to the historical hurricane interpreted to result in the deposition of each sand bed, although as noted in the Supplementary Information, individual sand beds could reflect storm deposition caused by multiple storms, and age model uncertainties prevent confident attribution of events to only one hurricane in many cases. (**a**) SLOSH modeled overwash regime (≥ 1.1 masl) storm tides at Shotgun Pond occurred during Category 1 storms in 1929, 1941, and 1966 (Alma), the Category 2 storms in 1856, 1886, and 1985 (Kate), Category 3 storms in 1877, 1894, 1896, 1926, 1975 (Eloise), 1985 (Elena), and 2005 (Dennis), and the Category 5 storm in 2018 CE (Michael). ADCIRC results^41^ indicate the storm tide for the Category 2 storm in 1852 CE surpassed the inundation regime threshold (≥ 5 masl). (**b**) SLOSH derived overwash regime (≥ 1.1 masl) storm tides at Basin Bayou occurred during the Category 1 hurricane in 1860, the Category 2 hurricane in 1936, and Category 3 hurricanes in 1917, 1926, 1985 (Elena), 1995 (Opal), and 2004 CE (Ivan). The modeled storm tides for the Category 3 hurricane in 1882 and the Category 2 hurricane in 1916 CE exceeded the inundation regime elevation at Basin Bayou (≥ 1.8 masl). (**c**) Comparison of the Bacon model (shaded) and ^210^Pb constant rate of supply model (not shaded) determinations of the age of the base of the event deposit at 21 cm (~ 1930 CE in panel **b**). Both age models overlap with the 1916 CE inundation regime hurricane (Supplementary Information).

Seventy-four tropical cyclones passed within a 150 km radius of Basin Bayou^6^ between 1851 and 2012 CE, the year of sediment core collection. The nine historic storms that produced modeled storm tides reaching overwash regime elevation (≥ 1.1 masl) were those with maximum sustained winds of at least 90 kts (Category 2 or greater) that made landfall west of Basin Bayou (Figs. 2, 3). Two of these storms, occurring in 1916 and 1882 CE, produced modeled storms tides reaching inundation regime at Basin Bayou (≥ 1.8 masl; Figs. 3, 4).

At Shotgun Pond, fifteen of the ninety-nine storms passing within 150 km resulted in modeled storm tides capable of flooding via the northern, low elevation route (≥ 1.1 masl; Figs. 1, 2). No SLOSH-modeled storm tides for events in the Best Track dataset exceeded the 5 masl inundation threshold at Shotgun Pond, however, the 1852 CE event exceeded the inundation regime threshold (> 5 masl) using more sophisticated Advanced Circulation (ADCIRC) modeling^41,42^. Only two hurricanes (1926 and Elena in 1985 CE) produced modeled overwash regime surges at both Basin Bayou and Shotgun Pond. These two storms tracked northwestward along the northern edge of the GOM such that Shotgun Pond and Basin Bayou were both in the onshore wind quadrants (i.e. front right) as the hurricanes passed by (Fig. 2). Other than these uncommon scenarios (< 3% of storms modeled for each site), historic hurricanes did not result in modeled flooding at both Basin Bayou and Shotgun Pond within the same event, so their respective reconstructions are likely to represent distinctly different storm histories. Overwash can occur at Shotgun Pond over a wider range of storm conditions compared to Basin Bayou, including tropical storm-strength cyclones, in part because of amplified storm surges related to coastally-trapped Kelvin waves in Apalachee Bay^41^. We expect that the sediment record from Shotgun Pond will record a greater number of storm deposits than from Basin Bayou due to its susceptibility to flooding under a wider range of storm conditions.

### Sedimentary records of storms

**Figure 5:**
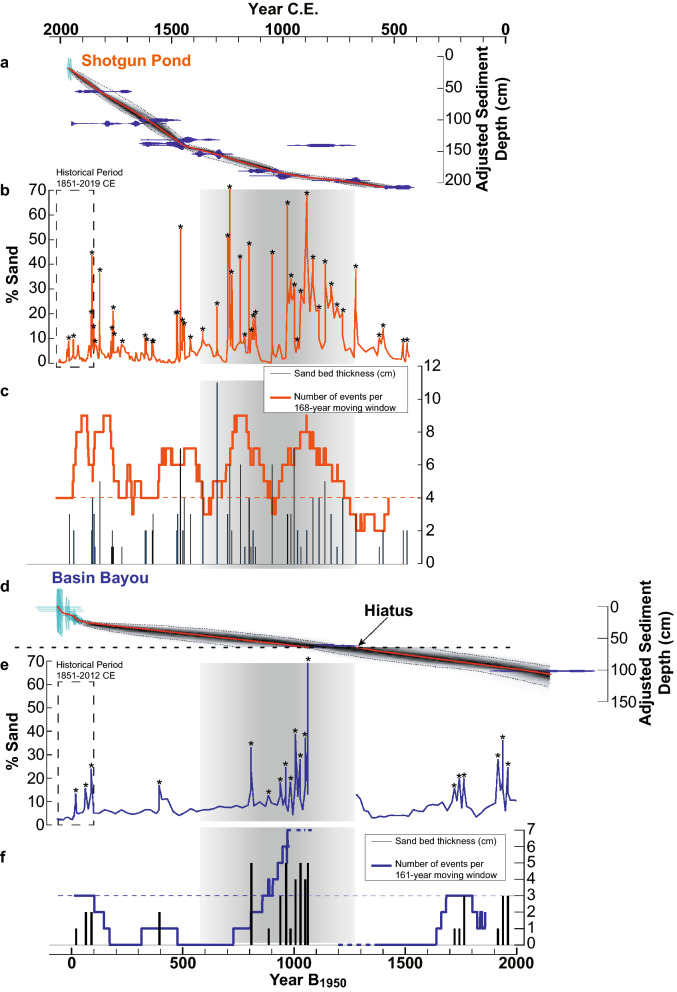
Age models and storm records from Shotgun Pond (**a**–**c**) and Basin Bayou (**d**–**f**). Age model outputs from Bacon^43^ are shown for Shotgun Pond (**a**) and Basin Bayou (**d**). The adjusted depth is the depth after sand bed removal. Darker gray shades indicate a higher density of age-depth profiles. The 95% confidence interval is indicted by the black dotted lines bracketing the age-depth shaded curves, the weighted mean age-depth profile is indicated by a solid red line, calibrated radiocarbon age probabilities are shown in blue, and ages derived from ^210^Pb and ^137^Cs are shown in green. In (**d**), a hiatus at 64.5 cm adjusted depth (102.5 cm composite depth with sand beds included) is indicated by a dashed horizontal line. A complete age model (4500 B_1950_ to present; B_1950_ = before 1950 CE)) for BaBy4 is displayed in Supplementary Fig. S1. Sand content is displayed as the percent of > 63 µm mass relative to the dry bulk mass (**b**,**e**), and sand beds removed prior to developing the age models (“events”) are indicated with an asterisk. The 168- and 161-year historic periods are outlined in black dashed rectangles for Shotgun Pond (**b**) and Basin Bayou (**e**), respectively. Thickness of sand beds in cm identified as “events” are displayed as vertical black bars for Shotgun Pond (**c**) and Basin Bayou (**f**). The number of events in 168- and 161-year moving windows are displayed in orange for Shotgun Pond (**c**) and blue for Basin Bayou (**f**), respectively. The horizontal orange (**c**) and blue (**f**) dashed lines indicate historical event frequencies, which is the historic baseline level relative to which “active” and “quiescent” intervals are discussed in the text. The bolded dashed blue line in (**f**) indicates the extrapolated number of events for portions of the record where the 161-year moving window is truncated by the depositional hiatus. Vertical gray shading highlights the active interval 650 to 1250 CE discussed in the text.

At Basin Bayou, a transect of sediment cores was collected in 2011 and 2012 CE perpendicular to the baymouth barrier separating it from Choctawhatchee Bay (Fig. 1; Supplementary Table S1; Methods). In this study, we focus on the long, continuous record from the sediment core located in the center of the bayou (BaBy4) to minimize influence of non-storm sand deposition related to Basin Creek discharge and baymouth barrier dynamics. At Shotgun Pond, a single sediment core was collected from the depocenter in 2008 CE (SHG1), and three surface cores were collected in 2019 CE. Supporting data from the supplementary cores from both sites are shown in Supplementary Figs. S2 and S3. For this study, we focused on the dark brown organic-rich very fine silt units characterizing the upper 3.7 m (~ 1500 years) at Shotgun Pond and the upper 1.5 m (~ 2000 years) at Basin Bayou, below which major lithologic changes indicate changes in the depositional environments that may alter susceptibility to storm overwash and preservation of overwash deposits in each of the sediment records (Supplementary Information). Age-depth models for each core were produced from^210^Pb (BaBy4 only), ^137^Cs, and ^14^C ages using Bayesian statistical analyses^43^ (Fig. 5, Supplementary Fig. S1), and storm deposits were detected using a combination of sieved sand fractions, geochemical analyses, and foraminifera identification (Methods). Modern sediment throughout Basin Bayou is characterized by very fine, organic-rich silt in a quiescent depositional environment. Sand is deposited in the bayou when it is entrained from the baymouth barrier and transported into and across the basin under high energy flood conditions, resulting in decreases in the thickness and frequency of sand beds observed in sediment cores with increasing distance from the baymouth barrier (Supplementary Information; Supplementary Fig. S2). Similarly, sand deposits punctuating the fine grained organic-rich background sedimentation in Shotgun Pond reflect storm-induced overwash deposits (Supplementary Information).

Historically, diagnostic storm deposits coincide with each of the Category 2 and 3 hurricanes that generated inundation regime storm tide maxima in the SLOSH model at both sites; three additional coarse deposits in Shotgun Pond and one additional deposit in Basin Bayou coincide with overwash regime storm tide (surge plus tide) maxima (Fig. 4; Supplementary Information). Hurricane Michael in 2018 did not cause flooding at Basin Bayou but resulted in overwash regime type flooding near Shotgun Pond^1^. This storm is represented by a ~ 5% increase in sand at the top of the 2019 sediment core (Fig. 4; Supplementary Fig. S3), which did not meet the event threshold (Methods). During either overwash or inundation regime floods at Basin Bayou, wave energy must be sufficient to transport sand nearly 700 m from the backside of the barrier to be preserved in sediments at the BaBy4 core location. Similarly, sufficient wave energy is required to transport sand roughly 2000 m from the Bald Point Peninsula shoreline inland to Shotgun Pond during inundation regime events, although sand can also be deposited into the pond during overwash regime events when storm surges funnel through the marsh on the north side of Bald Point. Consequently, only a few overwash regime floods during more intense and/or proximal storms are recorded at either site. The potential for erosion of the sediment record during inundation events, along with the approximately decadal sampling frequency of BaBy4 and subdecadal sampling frequency of SHG1, may limit these records from preserving the complete history of individual storm deposits, particularly those that occurred within a few months or years of one another (Supplementary Information). The sediment records from these sites thus capture only multi-decadal to centennial variability in the occurrence of intense hurricane landfalls.

Tropical cyclone activity varied substantially at the centennial timescale at both sites; both records contain multiple century-scale periods when sand content, event frequency, and sand bed thickness were greater than during the historic observational analog period (Fig. 5). At Shotgun Pond, the 168-year window spanning 1851–2019 CE was characterized by four storms deposits 2–3 cm thick and reaching 10–45% sand. The periods 650–1000, 1100–1300, 1350–1450, and 1750–1850 CE were characterized by higher storm frequency than the historic period (> 4 events per window), reaching 7–9 events per 168-year window. Storm deposits before 1500 CE contained a greater coarse fraction (up to 70% sand) and thicker (up to 11 cm) than those in the most recent six centuries (reaching 45% sand; 1–5 cm thickness). Quiescent intervals at Shotgun Pond (< 4 events per window), relative to 1851–2019 CE, were from 450–650, 1000–1100, 1300–1350, and 1500–1750 CE, where the period 450–650 CE was characterized by the fewest storms (2–3 per 168-year window), lowest sand contents (reaching up to 15% within each deposit), and thinner sand beds (1–2 cm).

At Basin Bayou, the 161-year period spanning 1851–2012 CE was characterized by three storm deposits 1–2 cm thick and reaching 13–24% sand. Storm frequency at Basin Bayou was comparable to modern from 0 to 300 CE, with both time periods averaging three events per 161-year window, sand content values reaching 13–36%, and sand bed thicknesses between 1 and 3 cm. The most active interval (> 3 events per window) of the Common Era at Basin Bayou spanned at least 900–1050 CE, reaching seven hurricane landfalls per 161-year window and storm deposit thicknesses of up to 5 cm with sand contents reaching up to 70%. An erosive event ~ 900 CE (Supplementary Information) resulted in missing record from 650 to 900 CE. Quiescent intervals relative to the historic period at Basin Bayou (< 3 events per window), relative to 1851–2012 CE, occurred from 250 to at least 650 CE and between 1150 and 1850 CE.

### Regional tropical cyclone histories

The storm reconstructions from Shotgun Pond and Basin Bayou share the same centennial-scale pattern of hurricane variability despite being sensitive to flooding by distinctly different individual storms. Unsurprisingly, the Shotgun Pond record indicates more intervals of heightened storm activity prior to the historic period relative to Basin Bayou, which is consistent with its susceptibility to flooding under a wider range of storm conditions. The period of greatest hurricane activity in both records occurred over multiple centuries centered on ~ 1000 CE, followed by a shift toward a prolonged quiescent period beginning around 1150–1300 CE (Fig. 5). Importantly, the historic period 1851 CE-present was characterized by reduced hurricane activity at both sites relative to ~ 1000 years ago, indicating that the short observational period is an underrepresentation of hurricane landfalls in the Florida panhandle.

**Figure 6:**
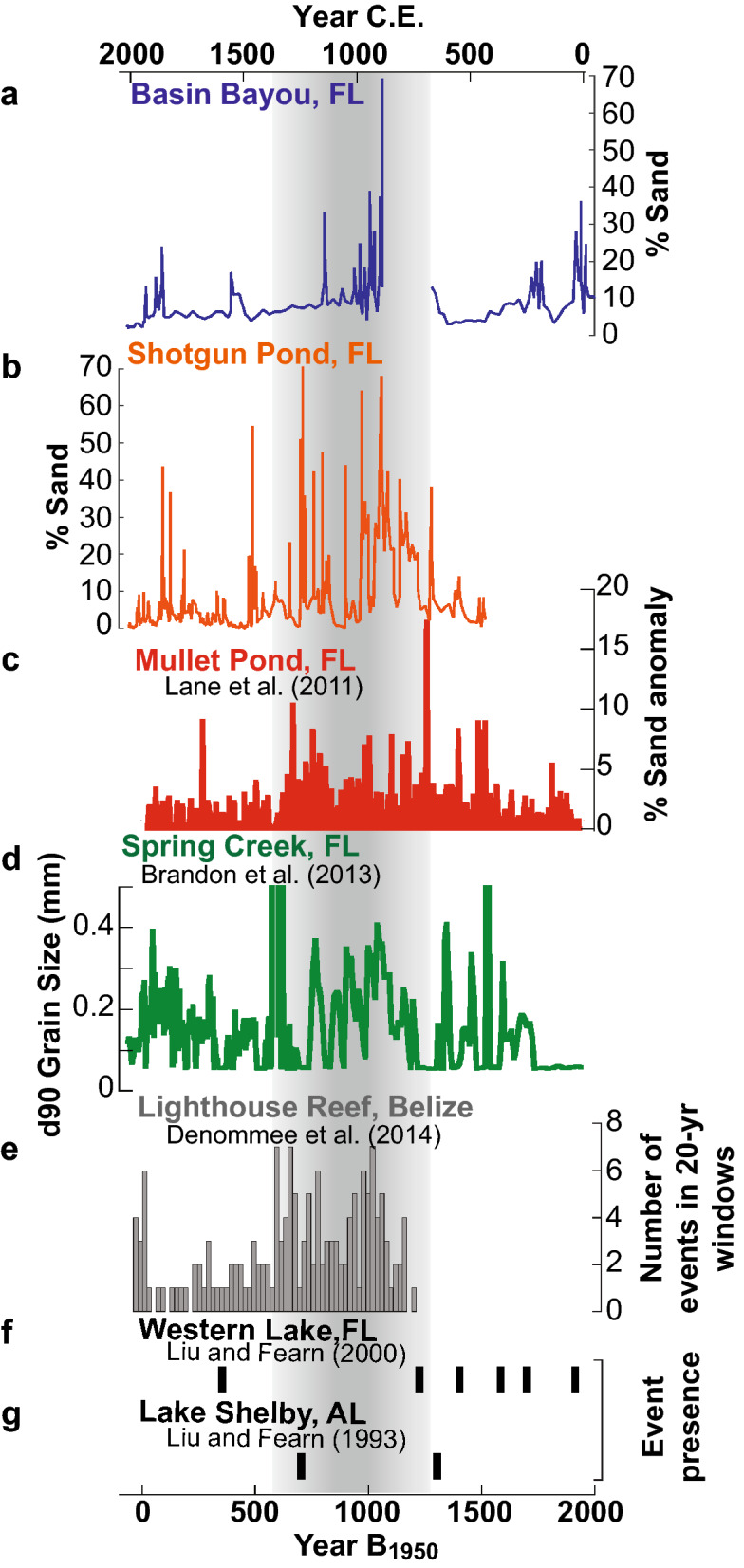
Comparison of paleostorm reconstructions from within the Gulf of Mexico and Belize. (**a**) Basin Bayou (blue; this study), (**b**) Shotgun Pond (orange; this study), **c.** Mullet Pond^36^ (red), (**d**) Spring Creek Pond^14^ (green), (**e**) Lighthouse Reef^46^ (gray), (**f**) Western Lake^35^ (black bars), and (**g**) Lake Shelby^34^ (black bars). Vertical gray shading highlights the same active interval (650–1250 CE) as in Fig. 5. B_1950_ = before 1950 CE.

The Basin Bayou and Shotgun Pond storm reconstructions are also similar to other grain size-based records of intense storms from the eastern panhandle region: Mullet Pond^36^, which is 200 km east of Basin Bayou and less than 1.7 km from Shotgun Pond, and Spring Creek Pond^14^, a ~ 2500-year-long reconstruction located 20 km north of Mullet Pond and Shotgun Pond (Fig. 1). These records together indicate that intense hurricane landfalls were more common along the northeastern GOM coast from 650 to 1250 CE relative to the historic period (Fig. 6). Most notably, hurricane activity decreased ~ 1150–1350 CE at each of these sites for 6–7 centuries. This shift toward decreased hurricane activity is consistent with a lack of intense hurricane deposits in sediments from Western Lake, FL^35^ and Lake Shelby, AL^34^ (Fig. 6). It should be noted, however, that the frequency and timing of storms recorded at Basin Bayou differs from that in Western Lake, despite their proximity. This discrepancy is perhaps related to an uncorrected reservoir effect on the radiocarbon-dated bulk sediment samples that form the Western Lake chronology, which was measured to be 985 years in nearby (< 6 km) Eastern Lake^44^. Radiocarbon ages forming the Basin Bayou chronology, on the other hand, were measured on terrestrial plant macrofossils (Table 1). Further investigation is needed to investigate the Western Lake chronology and resolve the differences between it and the Basin Bayou reconstruction. Evidence for a mid-millennium shift toward decreased hurricane activity extends beyond the northeastern GOM, including reductions in event deposits documented at Island Bay in southwest Florida^45^, Lighthouse Reef, Belize^46^ (Fig. 6), South Andros Island, The Bahamas^47^, Blackwood Sinkhole, The Bahamas^48^, and Laguna Playa Grande, Vieques, Puerto Rico^49^ (Fig. 1). Interestingly, a hurricane reconstruction from Salt Pond, Massachusetts^50^ documents increased hurricane activity for nearly three centuries during the GOM quiescent interval from 1400 to 1675 CE.

**Table 1:** Radiocarbon dating sample information for BaBy4 and SHG1 listed by cumulative depth in years before 1950 (B_1950_). CFAMS-dated samples are italicized. Samples included in the final age model for each core are marked with an asterisk next to the cumulative depth. Samples with a superscript “R” were rejected by Bacon.

Cumulative sediment depth (cm)	Lab sample code	Material dated	^14^C age (years B_1950_)	^14^C age error (years)
**Basin Bayou—BaBy4**				
64	Beta-467161	Plant Macrofossil	1180	30
85*	OS-102814	Plant Macrofossil	1190	35
153.5*	OS-102380	Plant Macrofossil	2140	65
*166.5*	*109,732*	*Bivalve*	*3118*	*156*
*176.5*	*109,733*	*Bivalve*	*3086*	*266*
*251*	*109,734*	*Bivalve*	*3502*	*266*
340*	OS-102189	Plant Macrofossil	4040	30
*501.5*	*109,735*	*Bivalve*	*5380*	*269*
**Shotgun Pond—SHG1**				
71*	OS-71340	Plant Macrofossil	105	15
129.5*	OS-146531	Plant Macrofossil	320	15
140*	OS-74404	Plant Macrofossil	220	25
165.5*	OS-146419	Plant Macrofossil	495	45
178*	OS-146420	Plant Macrofossil	355	20
188.5*	OS-146532	Plant Macrofossil	410	20
188.5^R^	OS-146421	Plant Macrofossil	1200	30
214*	OS-69596	Plant Macrofossil	675	25
225.5*	OS-146422	Plant Macrofossil	685	25
280*	OS-71341	Plant Macrofossil	955	20
305*	OS-69597	Plant Macrofossil	1050	25
338.5*	OS-146418	Plant Macrofossil	1320	25
353*	OS-74403	Bulk Sediment	1560	15

### Potential climate forcing

Cyclogenesis and storm maintenance rely on a number of factors including a steep temperature gradient between warm SSTs and the cold upper troposphere, a thick warm surface ocean layer to maintain SSTs and reduce cold water mixing from below, and minimal vertical wind shear to allow for deeper atmospheric convection^51^. We expect that low frequency ocean and atmosphere variability influence tropical cyclone development and strength on centennial-to-millennial timescales. SST variations within the GOM and the western Atlantic tropics likely influence GOM hurricane activity on these longer timescales akin to the shorter-term trends observed within the historical period^16^. Loop Current penetration in the GOM is also thought to be influenced by centennial-scale migrations of the ITCZ mean position^52,53^ with possible implications for GOM hurricane activity^14,15^. The mean position and strength of the North Atlantic subtropical high may influence the distribution of Atlantic hurricane landfalls at the centennial timescale for storms that formed in the MDR^10,12,16,50^, although historical data suggest the role of the subtropical high on directing the tracks of storms that formed within the GOM and western Caribbean Sea is insignificant^16^. Centennial-scale variations in the El Niño-Southern Oscillation system may also have influenced intense hurricane activity in the northeastern GOM records, though the histories of El Niño and La Niña are not clearly known. Here we discuss the relationship between GOM hurricane activity in the paleorecord and low frequency variability in factors that may have contributed to changes in the vertical thermal gradient and wind shear in the Atlantic and GOM regions in an effort to better understand the complex controls on intense GOM hurricane occurrence at centennial timescales.

**Figure 7:**
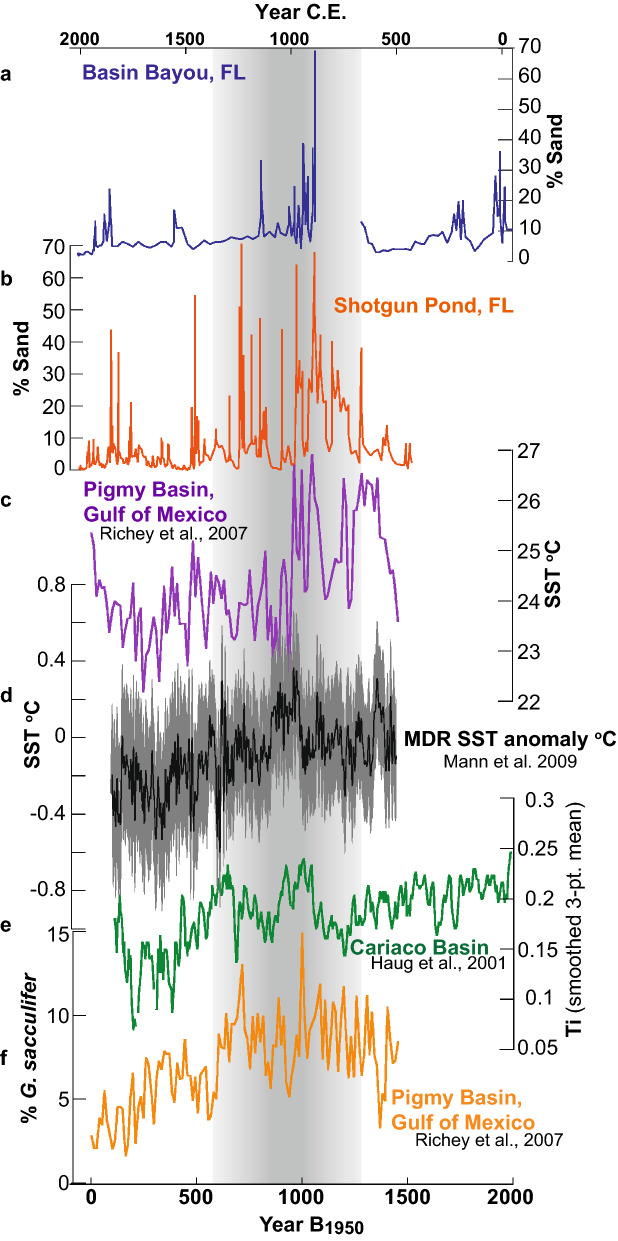
Comparison of GOM paleostorm reconstructions from (**a)** Basin Bayou (blue; this study) and (**b**) Shotgun Pond (orange; this study) plotted with (**c**) SSTs in the GOM Pigmy Basin^17^ (purple), (**d**) MDR SST anomalies (black) with the 2-sigma temperature range^12^ (gray), (**e**) Ti-inferred ITCZ variations in the Cariaco Basin^55^ (green), and (**f**) % G. sacculifer, a proxy for the Loop Current in the Pigmy Basin^17^ (orange). Vertical gray shading highlights the same active interval (650–1250 CE) as in Fig. 5.

Donnelly et al.^50^ identified warm SSTs in the northern tropics paired with a more northerly ITCZ as a potential mechanism controlling Late Holocene Atlantic basin hurricane activity, promoting cyclogenesis via a steepened thermal gradient and reduced wind shear. The mean position of the ITCZ migrates into the warmer hemisphere on decadal and longer timescales^54^ and should migrate and/or expand northward when the northern tropical SSTs warm, such as during periods of enhanced radiative forcing (e.g. the Medieval Climate Anomaly, or ‘MCA’ ~ 950–1250 CE). Runoff into the Cariaco Basin, inferred from sediment Ti concentration from the basin, is interpreted to reflect expansion and/or northward migration of the mean position of the ITCZ^55^, with increased Ti corresponding to a more northerly ITCZ (Fig. 7). Increased hurricane activity in the Atlantic Ocean during the MCA coincided with warmer SSTs in the MDR and a more northerly mean ITCZ in models^12^ and in paleorecords (e.g.^47–50^). Warm MDR SSTs and evidence for a more northerly ITCZ generally coincide with the MCA period characterized by more frequent intense tropical cyclone landfalls in Basin Bayou, Shotgun Pond, Mullet Pond, and Spring Creek Pond (Figs. 6 and 7). The reduction in GOM hurricane frequency during the Little Ice Age (LIA; ~ 1350–1800 CE) coincided with SST cooling in the MDR and evidence for a more southerly ITCZ (Fig. 7). Enhanced hurricane activity in the GOM during the MCA and reduced GOM activity during the LIA is consistent with Atlantic MDR SSTs and the ITCZ driving Atlantic basin hurricane activity.

Differences in hurricane patterns between the GOM and the Atlantic coast of the United States suggest factors influencing local cyclogenesis and/or storm maintenance may also play a role in Late Holocene hurricane activity, in addition to MDR SSTs and basin-wide convection and wind shear. For example, a recent reconstruction from Salt Pond, Massachusetts^50^ indicates that New England experienced an increase in landfalling hurricanes ~ 500 years before GOM hurricane activity increased (150 CE vs. 650 CE). Amplified hurricane formation and intensity along the New England and North Carolina coasts during the LIA, as indicated by an increase in event deposition in Salt Pond from 1400 to 1675 CE^50^ and more frequent inlet formation on the Outer Banks^56^, coincided with a quiescent interval in the northeastern GOM hurricane records. The Atlantic coast LIA active interval was attributed to a warm SST anomaly in the western North Atlantic Ocean^50^. During this LIA interval, GOM SSTs were also cooler compared to previous centuries^17,53^. These colder GOM SSTs may have inhibited cyclogenesis and/or weakened storms tracking into the GOM via a reduced thermal gradient and can explain why hurricane landfalls were less common in the northeastern GOM records from 1350 to 1850 CE.

Loop Current penetration into the northern GOM has previously been evoked as a mechanism explaining periods of heightened hurricane activity at Mullet and Spring Creek ponds (e.g.^14,15^. The extent of northward Loop Current penetration is closely tied to the position of the ITCZ^52^, so these factors are not necessarily independent. The ITCZ can therefore influence GOM hurricane activity by reducing wind shear and/or promoting Loop Current penetration into the northern GOM. A *G. sacculifer*-based reconstruction from the Pigmy Basin^17^ indicates that Loop Current penetration into the GOM was greater from 550 to 1350 CE, when GOM hurricane records indicate intense hurricane landfalls were more frequent (Fig. 7). On the other hand, a weaker Loop Current and cooler GOM SSTs during the LIA together may have contributed to a reduction in landfalling hurricanes in the GOM while hurricane activity was greater along the U.S. eastern seaboard. However, the concurrent decrease in hurricane activity during the LIA documented in the northeastern GOM sites and outside the GOM, including Belize, Puerto Rico, and The Bahamas, suggests that factors external to the GOM controlled hurricane activity over the past millennium. GOM hurricanes were perhaps locally amplified or weakened by the Loop Current in concert with Atlantic basin cyclogenesis.

Atlantic SSTs are projected to rise over the next century in response to greenhouse gas forcing (e.g.^57^), which may increase Atlantic basin hurricane activity, including in the GOM. If the future climate state is analogous to the MCA, despite differing forcing mechanisms driving surface warming, we expect more frequent and more intense hurricanes in the GOM than has been observed historically. On the other hand, model data suggests that the Loop Current will weaken over the next century, related to a slowing of Atlantic Meridional Overturning Circulation^58^, which leads to less warming in the GOM relative to the other oceans in models^23^. A weakened Loop Current and less pronounced GOM SST warming may inhibit GOM cyclogenesis and/or weaken cyclones forming within or entering the GOM, if intermediate waters in the GOM are cool enough to substantially reduce the vertical temperature gradient. A weaker Loop Current could thus lead to a reduction in intense hurricane activity in the northern GOM relative to the rest of the Atlantic basin, buffering the northern GOM from the predicted increase in Atlantic basin-wide intense hurricane activity. Yet, SSTs within the GOM are predicted to rise despite a weakened Loop Current^23^, which could fuel storm intensity. The degree to which enhanced tropical Atlantic and GOM SSTs, promoting cyclogenesis and stronger storms, is balanced by limited GOM SST warming and a weaker Loop Current, which limits storm formation and strength, remains unclear.

## Conclusions

We developed new records of hurricane landfalls in northwest Florida based on the identification of coarse deposits in sediment cores from Basin Bayou and Shotgun Pond. These new reconstructions documented multi-centennial variations in event frequency with heightened storm activity from 650 to 1250 CE relative to the last seven centuries (1250 CE to present). Enhanced hurricane activity in the GOM coincided with warmer SSTs in the MDR and within the GOM and evidence for a more northerly ITCZ and a stronger Loop Current at the multi-centennial timescale. The reduction in landfalling hurricanes circa 1250 CE ± 100 years is documented in storm reconstructions from multiple sites around the GOM and the Caribbean Sea and coincides with cooler SSTs in the MDR and within the GOM and evidence for a weaker Loop Current. Factors controlling Atlantic basin hurricane activity appear to modulate hurricane activity in the GOM at the centennial timescale given similarities between hurricane reconstructions within and external to the GOM. However, local factors that promote cyclogenesis within the GOM and/or influence the strength and duration of storms upon arrival in the GOM, such as GOM SSTs and Loop Current strength, are also important. Additional storm reconstructions from the GOM region, in particular records spanning several millennia, are necessary to evaluate these relationships at greater spatial and temporal scales.

While the future of hurricane activity in the northeastern GOM remains unclear, we present evidence for heightened hurricane activity during the last few millennia that exceeds levels observed from 1851 CE to present. Landfalling hurricanes were more common between 650 and 1250 CE relative to the past few centuries at multiple sites along the northeastern GOM coast. Consequently, the observation period 1851 CE to present does not represent the full range of natural variability in GOM hurricane activity and provides an incomplete baseline for determining whether landfalls of intense storms like Hurricane Michael are unusual in the context of past storm activity.

## Methods

### Storm surge modeling

Historical tropical cyclone data for our study sites are from the International Best-Track Archive for Climate Stewardship (IBTrACS) dataset obtained from the National Ocean and Atmospheric Administration Coastal Services Center^6^. We simulate flood heights across a range of storm proximities and intensities using the Sea, Lake, and Overland Surges from Hurricanes (SLOSH) model^59^ to estimate the vulnerability of our study sites to historical storm-induced flooding (Supplementary Table S2). SLOSH uses the barometric pressure difference across the radius of the storm, estimated from maximum wind values from the Best-Track historic storm data, to approximate storm intensity^59^**.** Radius of maximum wind (RMW) observations are often missing from historic storm track datasets, only becoming commonly available for storms occurring after 1995. For storms occurring prior to 1995, the RMW was estimated using multiple linear regression analysis to identify the relationship between storm radius, latitude, and observed maximum wind speed, similar to Quiring et al.^60^. Using storm radii observations included with the National Oceanic and Atmospheric Administration (NOAA) Extended Best Track dataset^61^, this analysis yielded the relationship:

$${\text{RMW }} = { 47}.{79 } + \, 0.{38831}\left( {{\text{Lat}}} \right) \, {-} \, 0.{35753}\left( {{\text{Vmax}}} \right)$$

where “Lat” is the latitude of each storm observation and “Vmax” is the observed maximum wind speed at that observation. To account for the stochasticity of hurricane development and radii beyond the simple linear approach above, each storm was modelled using the calculated RMW as well as ± 1σ uncertainty (Fig. 3). The maximum RMW modelled was 97 km (Agnes, 1972 + 1σ). Of the > 8000 storm observations that contain radii values in the Extended Best Tract Dataset, 776 exceeded a radius of ~ 100 km. A minimum RMW was set at 16 km since few (*n* = 42) of the storm observations in the Extended Best Track data set fall below that value.

Storm surge outputs generated by SLOSH were added to tide predictions to estimate the storm tide coinciding with each storm’s nearest pass to Basin Bayou. Tide predictions were obtained from the NOAA tide predictions tool at Valparaiso, FL (station ID: 8729501^62^), 23 km east of Basin Bayou on the north coast of Choctawhatchee Bay. Events occurring after 1923 were compared with available recorded storm tide levels captured by the tide gauge in Pensacola (station ID: 8729840^62^) to check the veracity of the model. Astronomical tides were not considered at Shotgun Pond due to the small tide range of ± 0.2 m^41^. Our approximations of storm tide lack wave height simulations at both sites and consequently may underestimate the true storm tide.

We use Lidar elevation data^63^ to approximate the surge height threshold at which each site floods given the modern site configuration. We identify historical storms that produced modeled minimum surge heights necessary to flood Basin Bayou and Shotgun Pond to understand which storm conditions tend to produce flooding at these sites and to compare with the historic portion of the sediment records.

### Sediment core collection

Our storm reconstructions are based upon lithologic changes in sediment cores from Basin Bayou and Shotgun Pond (Fig. 1). We collected sediment cores in 2011 and 2012 from nine locations in Basin Bayou in a transect roughly perpendicular to the baymouth barrier (Fig. 1; Supplementary Table S1). The surface sediments from each site were collected with a piston corer, to better preserve the less consolidated upper meter of sediments. Long vibracores and the overlapping surface drives from separate cores were combined into a composite core from BaBy3, BaBy4, BaBy5, BaBy6, BaBy8, and BaBy9 by matching visually distinctive bedding and trends in geochemical data. A vibracore and an overlapping surface piston core were taken from the deepest part of Shotgun Pond in 2008 (SHG1)^15^, and the surface piston core was replicated in 2019 (SHG1-MC-D1; Fig. 1; Supplementary Table S1). The 2019 surface drive was stratigraphically correlated with the 2008 cores using diagnostic variations in clastic sand and organic contents (Supplementary Fig. S3). A Pearson correlation demonstrates that the percent sand values from each core are significantly positively correlated after a ~ 4 cm adjustment to account for sediment accumulation between 2008 and 2019 (*p* < 0.01). The cores were split and described using the classification method from Schnurrenberger et al.^64^.

### Sedimentary analyses

We measured the sand content from Shotgun Pond by sampling the core at continuous 1 cm increments, drying the sediment samples at 105 °C for 24 h, burning the dried sediment and combusting organics at 550 °C for 2 h (2008 cores) or 4 h (2019 cores), and sieving the remaining inorganic ash through a 63 µm sieve. The samples were weighed after each step to obtain water, organic, and sand content, respectively^65^. We obtained sand contents from Basin Bayou sediments using a modified version of this procedure, because the clay-rich sediments became too hard to sieve following the drying and loss-on-ignition (LOI) steps. We separated each sample from Basin Bayou into two subsamples and performed LOI procedures on one subsample and sieving procedures on the other subsample. One subsample was dried overnight in a convection oven at 105 °C to determine the water content and combusted in a muffle furnace at 550 °C for four hours to determine the organic content (% LOI). The other subsample was wet-sieved at 32 µm to remove the fine particles that fuse together when the samples are dried. The sieved subsamples were then combusted in a muffle furnace at 550 °C for four hours to remove all coarse organic material^65,66^ and wet-sieved at 32 and 63 µm post-combustion to determine the coarse silt and sand contents. Sand content measured with this type of LOI and sieve procedure is typically reported in % greater than 63 µm relative to the bulk dry mass of the sample (e.g.^36^); the bulk dry masses of the sieved subsamples were approximated using the initial wet weight of each subsample before sieving and the water fraction determined on the other subsample from the same depth. The fraction of sediment greater than 63 µm relative to the bulk dry mass is referred to as % sand in this manuscript.

A 30-cm section of SHG1 (269–299 cm) that displayed prominent sand layers was sampled in approximately 1 cm increments for foraminiferal analysis (Supplementary Table S3). Surface and near surface samples (0–1 and 2–3 cm) in SHG1 were used to establish the present foraminiferal species assemblage in the pond. The foraminifera were concentrated by rinsing each ~ 3 cm^3^ sample through sieves, and the fraction of sediment between 500 µm and 32 µm in diameter was collected and analyzed for foraminiferal abundances. Identification and distribution relations were established from refs.^67–70,70^ and the world register of marine species (https://www.marinespecies.org/index.php).

### Core chronology

The upper 40 cm of sediments in BaBy4 were sampled every 3 cm, dried overnight in a convection oven at 105 °C, and homogenized with a mortar and pestle for gamma counting to obtain ^210^Pb and ^137^Cs activities. The upper 23 cm in SHG1 were sampled continuously in 1-cm intervals to measure the ^137^Cs activity profile. ^210^Pb and ^137^Cs profiles were measured on gamma detectors at Woods Hole Oceanographic Institution. We used ^137^Cs profiles in BaBy4 and SHG1 to identify the sediment horizons that corresponded with the onset of nuclear weapons testing (~ 1954 CE) and peak atmospheric ^137^Cs levels in 1963 C.E.^71^ (Supplementary Fig. S5). Unsupported ^210^Pb activities in the upper sediments from BaBy4 were used to construct a constant rate of supply (CRS) model for the last century^72,73^. Unsupported ^210^Pb activity values were determined by subtracting the background activity, assumed to be the average of the activities below the depth where ^210^Pb activity no longer decreased with increasing depth, from each ^210^Pb activity measurement.

Age information below the ^210^Pb profile in Basin Bayou sediments is from a combination of ^14^C ages on intact bivalve halves using the Continuous-Flow Accelerated Mass Spectrometer (CFAMS) method at the National Ocean Sciences AMS facility^74^ and organic ^14^C ages on plant macrofossils that were strategically sampled near major sedimentological transitions detected in radiographic images (Table 1). The ages derived from bivalves had large age uncertainties, exceeding several centuries, and were excluded for the development of the core chronologies due to the potential for an unknown reservoir effect. A single age derived from a plant macrofossil that was inadvertently sampled from within an event bed at 64 cm depth (Supplementary Fig. S2) was also excluded from the core chronology due to the high potential for reworking of older material within event beds. Plant macrofossils near, but outside of, event beds were prioritized for inclusion in the age model, although few well-preserved plant macrofossils of sufficient size for radiocarbon dating were available between event beds. Eleven ^14^C ages obtained from plant macrofossils and one ^14^C age from a bulk sediment sample were used for age control on SHG1 below the ^137^Cs profile (Table 1).

Age modeling for this study was completed using the IntCal13 curve to calibrate radiocarbon ages^75^ and version 2.2 of the Bacon age modeling software, which uses Bayesian statistics to compute weighted mean ages and age uncertainties for each 1-cm interval in the core^43^. Prior to age modeling, we removed sediment beds interpreted to reflect “instantaneous” deposition events and subsequently reinserted them following the age-depth estimation. We first applied a core-top chronology using the ^210^Pb CRS model (BaBy4) and ^137^Cs activities (SHG1) to identify the section of core representing the historic period 1851-present. We distinguished storm deposits from background variations in sand content by identifying sand content values exceeding the 80th percentile value for the historic period (≥ 11.7% at Basin Bayou; ≥ 7.8% at Shotgun Pond), a method similar to that in other paleohurricane reconstructions (e.g.^36,50^). These deposits and those meeting the same criteria deeper in the core, prior to the historic period, were removed prior to age-depth modeling and subsequently reinserted as instantaneous events. Supporting age information from radiocarbon dates measured on plant macrofossils and bivalves collected from cores BaBy1, BaBy6, BaBy8, and BaBy9 are listed in Supplementary Table S4 and displayed in Supplementary Fig. S2.

Sediment accumulation at both sites was relatively constant in the units analyzed for storm deposition, with considerably higher average sedimentation rates in Shotgun Pond (2.3 mm yr^-1^) relative to Basin Bayou (0.7 mm yr^-1^). In the surface sediments of BaBy4, sedimentation rates averaged 2.6 and 2.3 mm yr^-1^ based on the ^137^Cs activity peak (12.5 cm) and the ^210^Pb CRS model, respectively (Supplementary Fig. S5). In SHG1, the surface sedimentation rate averaged 4.3 mm yr^-1^, based on the rise and peak in ^137^Cs activity at 23 and 19 cm, respectively. All calibrated ages are reported in years in the Common Era (CE).

### Tropical cyclone deposit detection and frequencies

To compute storm deposit frequencies for the Common Era, we counted the number of sand beds exceeding the 80th percentile for sand content over the historic period; these are the same sand beds removed prior to age-depth modeling as described in the Core Chronology Methods above. This method isolated three distinct storm deposits in the 161-year historic periods at Basin Bayou (1851–2012 CE) and four storm deposits in the 168-year historic period at Shotgun Pond (1851–2019 CE; Fig. 4). We summed the number of events in 168-year and 161-year moving windows for the entire record from Shotgun Pond and Basin Bayou, respectively (Fig. 5), to best compare with event deposit frequencies calculated using similar methods in other paleostorm records (e.g.^36,50^). We also computed sand deposit thickness for each event identified with these methods, rounded to the nearest centimeter. This approach allows us to compare past storm deposition characteristics relative to the historic observational analog period in each core.

## Supplementary information

Supplementary Information

## Data Availability

The datasets generated during and/or analyzed during the current study are available in the National Centers for Environmental Information Paleoclimate repository, https://www.ncdc.noaa.gov/data-access/paleoclimatology-data, and in Supplementary Information files.
