# Supplementary material for: Historically unprecedented Northern Gulf of Mexico hurricane activity from 650 to 1250 CE
Source: Sci Rep. 2020 Nov 5;10:19092. doi: 10.1038/s41598-020-75874-0 (PMC7645782; doi:10.1038/s41598-020-75874-0)
Supplement: Supplementary file 1 — Supplementary Information [file 41598_2020_75874_MOESM1_ESM.pdf]

Supplementary Information

Historically unprecedented Northern Gulf of Mexico hurricane activity from 650 to 1250 CE

Jessica R. Rodysill[1, 2, 3], Jeffrey P. Donnelly[2], Richard Sullivan[2,4], Philip D. Lane[†], Michael Toomey[1, 2], Jonathan D. Woodruff[5], Andrea D. Hawkes[2,6], Dana MacDonald[2,5] Nicole d'Entremont[2], Kelly McKeon[2,5], Elizabeth Wallace[2], Peter J. van Hengstum[2,4,7]

[1]Florence Bascom Geoscience Center, United States Geological Survey, Reston, VA
[2]Geology and Geophysics, Woods Hole Oceanographic Institution, Woods Hole, MA
[3]Department of Earth, Environmental and Planetary Sciences, Brown University, Providence, RI
[4]Department of Oceanography, Texas A&M University, College Station, TX
[†]Deceased
[5]Department of Geosciences, University of Massachusetts, Amherst, MA
[6]Department of Earth and Ocean Sciences, University of North Carolina Wilmington, Wilmington, NC
[7]Department of Marine Sciences, Texas A&M University, Galveston, TX

Sediment lithology and basin evolution

Basin Bayou

The sediments from Basin Bayou gradually transition from very coarse quartz sand, in the bottom of BaBy4, BaBy6, and BaBy8, to a 2 to 5 m-long unit composed of medium gray silty clay, to dark brown very fine organic-rich silt in the upper 1.5 to 2 m of each core, corresponding to a gradual increase in organic matter (% LOI). Several 0.2 to 3 cm-thick beds characterized by sand content increases of up to 87% occur throughout each core (Supplementary Figure S2). In the lower gray silty clay unit, coarse beds are composed of quartz sand with broken pieces of carbonate shells and, in some cases, very coarse (> 10 mm) terrestrial plant material. In the upper organic-rich silt unit, the beds do not contain carbonate shell fragments. An abrupt geochemical and lithological transition at a sharp erosional contact is apparent in each sediment core (Supplementary Figure S2); these observations, paired with chronological information (Table 1 and Supplementary Table 4), led us to infer a depositional hiatus at 102.5 cm (~900 CE) in BaBy4. The timing of the bayou-wide hiatus roughly coincides with evidence for the opening of the East Pass in the barrier separating Choctawhatchee Bay from the Gulf of Mexico, which is hypothesized to be caused by an intense storm[76].

The lithologic transitions in Basin Bayou cores described above are clear evidence of past changes in the depositional environment. The gray clay in all bayou cores below 1.5-2 m core depth (~2000 B_{1950} in BaBy4) is macroscopically identical to surface sediments in Choctawhatchee Bay. Modern Choctawhatchee Bay sediments also contain abundant carbonate shells, similar to the carbonate shell fragments observed in coarse beds within the lower gray clay unit in Basin Bayou sediments. These shell fragments are absent in the upper 1.5-2 m of Basin Bayou sediments; the transition to coarse beds that lack shell fragments coincides with the transition to sapropel. We infer that the connection between the bayou and Choctawhatchee Bay was more open prior to 2000 B_{1950}, similar to the two embayments east of Basin Bayou (Figure 1). The lithologic transition in Basin Bayou just prior to 2000 B_{1950}, diverging from the Choctawhatchee Bay facies, likely indicates that the bayou became more isolated from

the bay as baymouth barrier evolution weakened the connection between the two water bodies, which has implications for the sensitivity of the site to hurricane overwash.

During the mid-Holocene, when sea level was ~4 m lower than present[77], the distance between the Choctawhatchee Bay and Basin Bayou would have been >2000 meters greater than present. After 2000 B_{1950}, sea level was likely within 1.5 m of mean sea level[77]. A greater distance in the early portion of the record, prior to 2000 B_{1950}, would necessitate higher wind-driven transport energy to deliver sand laterally to BaBy4 relative to modern. Barriers like the baymouth barrier are transient landforms that are sensitive to sediment availability and changes in water current direction and strength[78]; late Holocene sea-level rise likely altered bay currents such that increased sediment supply constructed and expanded the baymouth barrier[79]. Barrier expansion likely increased the overwash and inundation regime elevations, reducing the sensitivity of Basin Bayou to storm deposition as evidenced by the disappearance of carbonate shell fragments in coarse deposits after ~2000 B_{1950}. However, an expanded barrier is an important proximal source of unconsolidated sand that could be more readily transported into the bayou, potentially resulting in more numerous and/or thicker sand beds without necessitating a change in storm climatology. A decreasing site-to-sea distance could also make the site more vulnerable to storm deposition compared to the mid-Holocene. By focusing this study on the past 2000 years, we attempt to minimize the effects of changing site sensitivity on the preservation of tropical cyclone landfalls in the sediment record.

Shotgun Pond

The lower 0.3 m of SHG1 is composed of limestone debris, which is overlain by about a meter of coarse sand. These sediments have been interpreted to reflect initial sediment infill following sinkhole collapse[15]. The upper 3.7 m of SHG1 is a dark brown fine silt with algal and vascular organic matter interbedded with cm-scale sand beds and is the section that was analyzed for storm deposition[15]. Major lithologic changes are indicative of a changing depositional environment, and the pond sediments may not record storm deposition consistently from one lithologic unit to the next. For this reason, we focused only on the sapropel unit for our reconstruction. Consistency in sand content variations between the upper

meter of the sediment cores collected in 2008 and 2019 demonstrate that the event stratigraphy is replicated in both the north and southeast sections of the pond (Supplementary Figure S3).

Foraminiferal species diversity ranged from one to six, when present, and all species had agglutinated test forms (*Ammobaculites dilatatus, Ammobaculites exiguus, Arenoparrella mexicana, Haplophragmoides* sp., *Miliammina fusca*, *Lagenammina atlantica*); no calcareous forms were found. Zero to 144 individuals were identified from the 19 samples between 269-295 cm depth in SHG1 with the highest diversity at 279 and 298 cm (5 species). The presence of foraminifera coincides with increases in sand content. Foraminifera were absent from seven depth horizons (2.5, 274.5, 275.5, 276.25, 276.75, 277.25, 285.5 cm) that do not show an increase in sand content. In the surface sample at 0-1 cm only *Haplophragmoides* sp. were present in very low abundance (Supplementary Table S3). Low species abundance (4-16 individuals) was also observed in six additional samples (269.5, 272.5, 278.75, 282.5, 286.5, 287.5).

During the ~1500-yr record of storm deposition, sea level in the GOM was within ~1 m of mean level[77], and the presence of brackish foraminifera in nearby Mullet Pond indicate a transition from fresh to brackish water and development of a salt marsh by ~2400 B$_{1950}$[36], nearly a millennium before the start of the Shotgun Pond storm reconstruction. The Shotgun Pond storm reconstruction thus captures storm activity over a period of relatively stable sea level, similar to Basin Bayou.

Coarse sediment source and transport mechanisms

Basin Bayou

Sand beds in the BaBy4 sediment core can be derived from the baymouth barrier when storm surges in Choctawhatchee Bay exceed the elevation of the baymouth barrier, energetic waves that transport sand through the inlet, and flooding in the Basin Creek drainage and/or the catchment surrounding the bayou due to runoff during heavy rainfall. The transect of sediment cores along the long axis of Basin Bayou was strategically collected to determine the relative contributions of sand from Basin Creek and the baymouth barrier. The frequency of sand beds identified in each core and the amplitude of

the increase in sand content in individual beds decrease with increasing distance from Choctawhatchee Bay (Supplementary Figure S2). Floods in the small drainage basin of Basin Creek [39] are unlikely to be significant contributors to forming basin-wide sand sheets, and the sparsity of sand beds and low sand values in the core proximal to the mouth of the creek, BaBy9, supports our interpretation. Runoff from the very flat (< 1m/km) catchment is also unlikely to transport enough sand to form the thick, cm-scale sheets of sand across the bayou that are observed in the transect of cores, though some littoral sand deposition is possible. We expect that a greater amount of sand is deposited closest to its source, implying that the majority of basin-wide, thick sand beds are derived from the transport of sediment from the baymouth barrier to the BaBy4 core site.

Tropical cyclones capable of inundating the baymouth barrier are (1) those that make landfall to the west of the site, producing winds mainly in the onshore direction such that the storm surge propagates through the opening on the southwest side of Choctawhatchee Bay and then eastward within the bay, (2) storms that have tracked to the north side of Choctawhatchee Bay, driving strong westerly winds that produce large-amplitude waves that propagate eastward across Choctawhatchee Bay, and (3) those that make landfall directly over our site such that the maximum wind velocities pass directly over the Basin Bayou, producing waves that overtop the baymouth barrier (Figures 2, 3). Given the long fetch of Choctawhatchee Bay, storms of low intensity that produce westerly winds may be capable of producing wind-driven surges and/or waves within Choctawhatchee Bay that overtop the barrier.

Tropical cyclones producing storm tides that reach overwash regime elevations but do not completely inundate the barrier are likely to result in sand deposition limited to the southern end of Basin Bayou nearest the low elevation runoff flows over the baymouth barrier. During events where storm tide falls within the collision regime (up to 1.1 masl), sand may be transported by large waves that pass through the inlet from Choctawhatchee Bay, at the south end of the bayou. During events where storm tide ranges fall within the overwash regime (1.1-1.8 masl), sand may also be transported by large waves that overtop the baymouth barrier and are funneled through low elevation channels on the barrier. During

events where storm tide exceeds 1.8 m (inundation regime), the barrier and bayou are completely submerged, resulting in sheet overwash and widespread sand deposition.

Shotgun Pond

Sand beds in the upper 3.7 m of SHG1 could be formed by sediment focusing within the pond during stormy conditions or when unconsolidated Holocene and Pleistocene-aged sand from the Bald Point Peninsula is transported into the pond. Allogenic sand may be entrained in runoff within the small catchment during periods of heavy precipitation, subaerially-transported into the pond in strong winds, or transported from outside the catchment during floods. Reworking and redeposition of sandy shoreline material in the depocenter is highly unlikely given the small fetch of the pond relative to water depth, and wind-driven sand deposition is unlikely to result in the 1-11 cm-thick sand beds observed throughout the core (Figure 5), especially considering that most of the catchment is stabilized by vegetation. Flood deposition is a more likely source of thick sand beds in the center of the pond.

In the 30 cm interval that was analyzed for foraminifera content, the two sand beds, centered at ~280 and ~290 cm, are associated with higher abundances of foraminifera relative to the surrounding sediment (Supplementary Figure 4; Supplementary Table S3)[15]. These foraminifera, including *Ammobaculites dilatus* and *Ammobaculites exiguus,* typically live in shallow marine, open coast siliciclastic environments, and indicate that the source of sediment within those beds was at least partially derived from a marine setting[80]. We note that *Ammobaculites* also live in brackish environments such as tidal flats, low marsh, and coastal ponds; the absence of these foraminifera in the core-top sediments at Shotgun Pond suggests that sand beds containing *Ammobaculites* are delivered into the pond from the surrounding bays and/or tidal marshes during high energy conditions. The presence of a few *Haplophragmoides* sp. in the surface sample of SHG1 could suggest that the pond is now slightly brackish because of rising relative sea level. *Haplophragmoides* sp. are often found in the lower salinity high and middle salt marsh zones [81]. Offshore surface sample transects in the GOM from ref. [36] show agglutinated species *A. dilatatus, A. exiguus,* and *M. fusca* present in nearshore samples between 1-10 m water depth, and the shoreface (1 m water depth to 3 masl) is barren of all foraminifera. Many calcareous

species are also present from 1 to 15 m water depth in the GOM (the deepest the study sampled), but none were recovered in SHG1. Either calcareous species were absent from transport, or, more likely, they suffered from post-depositional dissolution once subjected to the pond environment. The presence and often increased diversity of agglutinated foraminifera coinciding with increased sand content and the near total absence of any foraminifera in samples lacking increased sand content suggests that the foraminifera were transported into Shotgun Pond. Alternatively, the brief increase in salinity from overwash during storms caused the pond to be environmentally hospitable to agglutinated foraminifera for these punctuated periods, however, increased rainfall during storm events often causes freshening. Foraminifera were most likely transported to Shotgun Pond during storms, and calcareous forms were impacted by taphonomic processes. These observations support the storm surge and inundation model results indicating that this site is vulnerable to flooding by storm surges that are capable of transporting coarse material over 2.3 km from the Gulf Coast into the pond[41]. The presence of these shallow marine foraminifera from over 1000 years ago suggests that this site has been vulnerable to storm-induced flooding through at least the past millennium. The ~1 m of sea-level rise that occurred over the short duration of this record[77] is unlikely to significantly control the sensitivity of Shotgun Pond to storm-induced deposition.

Storm-driven coarse sediment deposition is possible in Basin Bayou and Shotgun Pond when the local storm surge exceeds 1.1 m. Additionally, substantial wave energy capable of transporting sand for hundreds of meters is necessary to deposit the thick sand beds observed in the Basin Bayou sediment cores that are far from the shoreline or to transport open coast foraminifera inland to Shotgun Pond. Wave energy strongly controls the grain size of the particles that are transported. Woodruff et al.[82] demonstrated with an advective settling model that sand-sized grains can be transported ~200 m under Category 5 storm strength winds at Laguna Playa Grande, Puerto Rico. While this model is not directly applicable to Basin Bayou or Shotgun Pond, we can infer based on the fact that the BaBy4 core location is 3.5 times the distance from the barrier as the distance modeled at Laguna Playa Grande that intense wave energy would be required to deliver sand 700 m from the baymouth barrier to the BaBy4 core site.

Similarly, substantial flooding ($\geq$ 5 m) and high wave energy would be required to mobilize and transport sand ~2300 m from the beach dunes on the eastern shore of Bald Point to Shotgun Pond[41].

Historical storms preserved in Basin Bayou sediments

The Bayesian-determined 95% age uncertainty of the most recent storm deposit preserved in BaBy4 (20-21 cm composite depth; weighted mean age ~1930 CE) overlaps with three overwash regime events (1917, 1926, and 1936 CE) and one inundation regime event in 1916 CE (Figure 4). Although few Bayesian age model simulations align the sand bed with the inundation regime event, the ^{210}Pb age model indicates the base of this sand bed was deposited ~1916-1917 CE (Figure 4c). The storm deposit at 20-21 cm depth likely formed from the inundation regime hurricane in 1916 CE, and the subsequent sand beds that did not meet the event threshold (Methods) likely formed during the three subsequent overwash regime events.

Coarse deposit preservation potential in Basin Bayou and Shotgun Pond

Several factors influence the preservation potential of storm-induced coarse deposits, including rate of sedimentation, sediment transport processes within the depositional basin[82], and post-depositional mixing (e.g. bioturbation) of the sediments[83, 84]. The sedimentation rates in Basin Bayou are too low to resolve multiple storm deposits occurring within a few years of one another at our 1-cm (4-14 yr) sampling resolution. The modern sedimentation rate in Shotgun Pond is higher than in Basin Bayou, but our 1-cm (2-4 yr) sampling remains insufficient to distinguish two closely-spaced hurricane landfalls. Sand deposits, particularly thin beds, may be reworked due to surface bioturbation, resulting in vertical integration and dilution of coarse grains in the sediment column and reducing the magnitude and detectability of the coarse anomaly[83, 84]. The lack of fine laminae preserved in the sediments may be indicative of bioturbation, particularly in the upper organic-rich sediments at both sites. While bioturbation may dilute sand beds, the sharp lithologic contacts separating all coarse deposits are suggestive of minimal vertical mixing, if any. Storm deposits in Basin Bayou have the potential to be

reworked during subsequent storms and eliminated from the record in near-shore shallow core sites; subsequently, the reworked sediment can be deposited in the deepest part of the basin, near the BaBy4 site, such that multiple storm deposits are combined into a single sand bed.

The lack of a one-to-one relationship between historic sand beds and hurricanes with surges that exceeded the minimum flooding threshold implies that more hurricanes may have made landfall near these sites than is reflected by the frequency of sand beds in their sediment records. While sediment reworking, low sedimentation rate, and bioturbation may reduce the number of event deposits in our record relative to the true number of storms that made landfall near each site, there is no clear mechanism that is capable of producing basin-wide cm-scale sand beds other than tropical cyclone wind and wave energy. For these reasons, we assert that the basin-wide sand beds archived in Basin Bayou and Shotgun Pond are hurricane-induced and provide a conservative estimate of hurricane frequency.

Supplementary References

76. Ranasinghe, P.N., Donnelly, J.P., Evans, R.L., van Hengstum, P.J., Ashton, A.D., 2012. "Changes in hurricane activity in northeastern Gulf of Mexico during last 8500 yrs. – A hurricane record from Choctawhatchee Bay, Destin FL." American Geophysical Union Fall Meeting, San Francisco, CA, 3-7 Dec. OS31A-1699. Poster Presentation.
77. Törnqvist, T.E., Bick, S.J., van der Borg, K., de Jong, A.F.M., 2006. How stable is the Mississippi Delta? *Geology*. **34**, 697-700. doi: 10.1130/G22624.1.
78. Donnelly, J.P., Webb III, T., 2004. Backbarrier sedimentary records of intense hurricane landfalls in the northeastern United States. In: Murnane, R. and Liu, K. (eds.), Hurricanes and Typhoons: Past Present and Future, New York: Columbia Press, pp. 58-96.
79. Davidson-Arnott, R., 2010. An introduction to coastal processes and geomorphology. New York: Cambridge University Press.
80. Sen Gupta, B.K., Smith, L.E., Machain-Castillo, M.L., 2009. Foraminifera of the Gulf of Mexico. In Felder, D.L. and Camp, D.K. (eds.) Gulf of Mexico-Origins, Waters, and Biota: Biodiversity. Texas A&M Press, College Station, Texas. Pp. 87-129.
81. Hawkes, A.D. et al., 2016. Relative sea-level change in northeastern Florida (USA) during the last ~8.0 ka. *Quat. Sci. Rev.***142**, 90-101. doi:10.1016/j.quascirev.2016.04.016.
82. Woodruff, J.D., Donnelly, J.P., Mohrig, D., Geyer, W.R., 2008. Reconstructing relative flooding intensities responsible for hurricane induced deposits from Laguna Playa Grande, Vieques, Puerto Rico. *Geology*. **36**, 391-194. doi: 10.1130/G24731A.1.
83. Hippensteel, S.P., 2008. Preservation Potential of Storm Deposits in South Carolina Back-Barrier Marshes. *J. Coast. Res*. **24**, 594-601. doi: 10.2112/05-0624.1.
84. Hippensteel, S.P., 2010. Paleotempestology and the pursuit of the perfect paleostorm proxy. *GSA Today*. **20**, 52-53. doi: 10.1130/GSATG80GW.1.

Core Name	Year of collection	Latitude (decimal degrees)	Longitude (decimal degrees)	Core Length (m)	Water Depth (m)
BaBy1	2011	30.489733˚N	86.246317˚W	1.68	1.45
BaBy2	2011	30.489933˚N	86.245433˚W	1.31	1.45
BaBy3	2012	30.48792˚N	86.24368˚W	4.68	1.5
BaBy4*	2012	30.49213˚N	86.24368˚W	7.06	1.7
BaBy5	2012	30.4881˚N	86.24768˚W	4.37	1.4
BaBy6	2012	30.49033˚N	86.24995˚W	6.20	1.6
BaBy7	2012	30.48921˚N	86.24818˚W	2	0
BaBy8	2012	30.49671˚N	86.24207˚W	7.42	1.5
BaBy9	2012	30.50044˚N	86.24126˚W	4.64	0.6
SHG1*	2008	29.9316 ºN	84.355ºW	5.02	5.1
SHG1-MC-D1*	2019	29.93175ºN	84.35508ºW	1.77	5.7

Supplementary Table S1: Locations, lengths, and water depths for each core collected from Basin Bayou (2011, 2012) and Shotgun Pond (2008, 2019). Cores with an asterisk are discussed in detail.

Year [CE]	Name	Maximum sustained velocity during the lifetime of the storm [knots]	Category at landfall	Distance from Basin Bayou at landfall [km]	Landfall right (R) or left (L) of Basin Bayou	Radius [km]	1 σ (radius) [km]	Storm surge maxima (radius - 1σ) [m]	Storm surge maxima (radius) [m]	Storm surge maxima (radius + 1σ) [m]
1860	-	110	1	94.8	R	37.45	35.13	0.34	0.67	**1.13**
1867	-	90	1	124.6	L	49.57	35.21	0.15	0.09	0.06
1877	-	70	1	1.4	R	55.63	35.13	0.34	0.46	0.61
1887	-	85	1	25.4	R	53.29	35.13	0.55	0.7	0.7
1898	-	70	1	19	L	54.46	35.12	0.15	0.15	0.15
1903	-	80	1	46.2	L	49.91	35.13	0.12	0.12	0.12
1911	-	70	1	95.6	R	57.23	34.81	0.27	0.61	0.82
1915	-	85	1	83.2	L	49.63	35.13	0.18	0.06	0.06
1924	-	75	1	120.1	L	52.32	35.13	0.06	0.06	0.06
1929	-	135	1	60.8	L	58.31	35.13	0.12	0	0
1939	-	65	1	1.8	R	58.52	35.13	0.18	0.18	0.21
1953	Florence	100	1	17.2	L	54.57	35.13	0.27	0.58	0.98
1956	Flossy	80	1	3.13	R	60.66	35.13	0.18	0.34	0.46
1972	Agnes	75	1	93.7	L	61.73	35.13	0.09	0.09	0.09
1998	Earl	85	1	65.9	L	55.44	35.13	0.15	0.15	0.15
1856	-	100	2	46.3	L	51.56	29.71	0.15	0.09	0.15
1896	-	85	2	11.8	R	46.99	35.13	0.7	0.67	0.94
1899	-	85	2	82.3	L	46.56	35.13	0.24	0.18	0.15
1916	-	95	2	107.8	R	41.25	35.13	0.3	0.76	**2.5**
1936	-	90	2	36.8	R	44.13	35.13	0.4	0.79	**1.04**
1985	Kate	105	2	104.5	L	48.92	35.13	0.18	0.18	0.18
1995	Erin	85	2	75.6	R	32.19	-	-	0.12	-
1851	-	100	3	68.1	L	38.19	35.13	0.15	0.12	0.12
1877	-	100	3	97.2	L	38.13	35.13	0	0	0
1882	-	110	3	33.6	R	32.62	35.13	0.52	**1.28**	**2.41**
1894	-	105	3	68	L	35.37	35.13	0	0	0.06
1917	-	130	3	16.34	R	38.37	35.13	**1.04**	0.88	**1.4**
1926	-	130	3	67.6	R	35.78	35.13	0.12	0.61	**1.55**
1975	Eloise	110	3	1.1	L	32.49	35.13	0.12	0.37	0.76
1985	Elena	110	3	108.9	R	34.76	35.33	0.06	0.21	**1.49**
1995	Opal	130	3	69.2	R	40.23	-	-	**1.22**	-
2004	Ivan	145	3	152	R	31.86	-	-	**1.4**	-
2005	Dennis	130	3	80	R	37.18	-	-	0.64	-

Supplementary Table S2: Modeled and observed historical storm parameters and storm surge maxima at Basin Bayou. The hurricanes listed are Category 1 or stronger storms from the Extended Best-Track data set [6] that tracked within 150 km of Basin Bayou. Gray shading and bolded storm surge maxima indicate storms that produced modeled or observed storm surges exceeding 1 m above sea level at Basin Bayou. Storm radii and surges from observational data are underlined; the remaining radii and surges were modeled as described in Methods.

depth (cm)	*Ammobaculites dilatatus*	*Ammobaculites exiguus*	*Arenoparrella mexicana*	*Haplophragmoides sp*	*Lagenammina atlantica*	*Miliammina fusca*	Total number of individuals	Increase in sand
0.5	0.0	0.0	0.0	100.0	0.0	0.0	4	
2.5	0.0	0.0	0.0	0.0	0.0	0.0	0	
269.5	0.0	0.0	0.0	0.0	100.0	0.0	4	x
272.5	0.0	0.0	0.0	100.0	100.0	0.0	4	x
274.5	0.0	0.0	0.0	0.0	0.0	0.0	0	
275.5	0.0	0.0	0.0	0.0	0.0	0.0	0	
276.25	0.0	0.0	0.0	0.0	0.0	0.0	0	
276.75	0.0	0.0	0.0	0.0	0.0	0.0	0	
277.25	0.0	0.0	0.0	0.0	0.0	0.0	0	
278.75	0.0	100.0	0.0	0.0	0.0	0.0	8	x
279.5	8.3	50.0	0.0	27.8	11.1	2.8	144	x
282.5	25.0	0.0	25.0	50.0	0.0	0.0	16	x
285.5	0.0	0.0	0.0	0.0	0.0	0.0	0	
286.5	0.0	0.0	100.0	0.0	0.0	0.0	8	x
287.5	100.0	0.0	0.0	0.0	0.0	0.0	8	
289	33.3	55.6	0.0	11.1	0.0	0.0	36	x
291.5	0.0	70.6	23.5	5.9	0.0	0.0	68	x
294.5	0.0	12.5	12.5	75.0	0.0	0.0	64	x
298.5	10.0	15.0	10.0	55.0	0.0	10.0	80	x

Supplementary Table S3: Relative abundances of foraminifera identified in SHG1. The percent of foraminifera species per sample is listed relative to the total number of individual foraminifera counted in each sample analyzed for microfossil abundances. The depth listed in the first column is the center depth of each 1 cm sampling interval. Samples with increased sand content are indicated by an ‘x’ in the last column.

Cumulative sediment depth (cm)	Lab sample code	Material Dated	^{14}C age (years B_{1950})	^{14}C age error (years)
**BaBy1**				
48.25	OS-98130	Plant Macrofossil	365	25
122.3	*OS-96543*	*Bivalve*	*1430*	*100*
**BaBy6**				
71.9	OS-102190	Plant Macrofossil	1920	25
98.65	OS-102403	Plant Macrofossil	2190	30
**BaBy8**				
128.3	OS-135390	Plant Macrofossil	2690	20

Supplementary Table S4: Radiocarbon dating sample information for BaBy1, BaBy6, and BaBy8 listed by cumulative depth in years before 1950 (B_{1950}). CFAMS-dated samples are italicized.

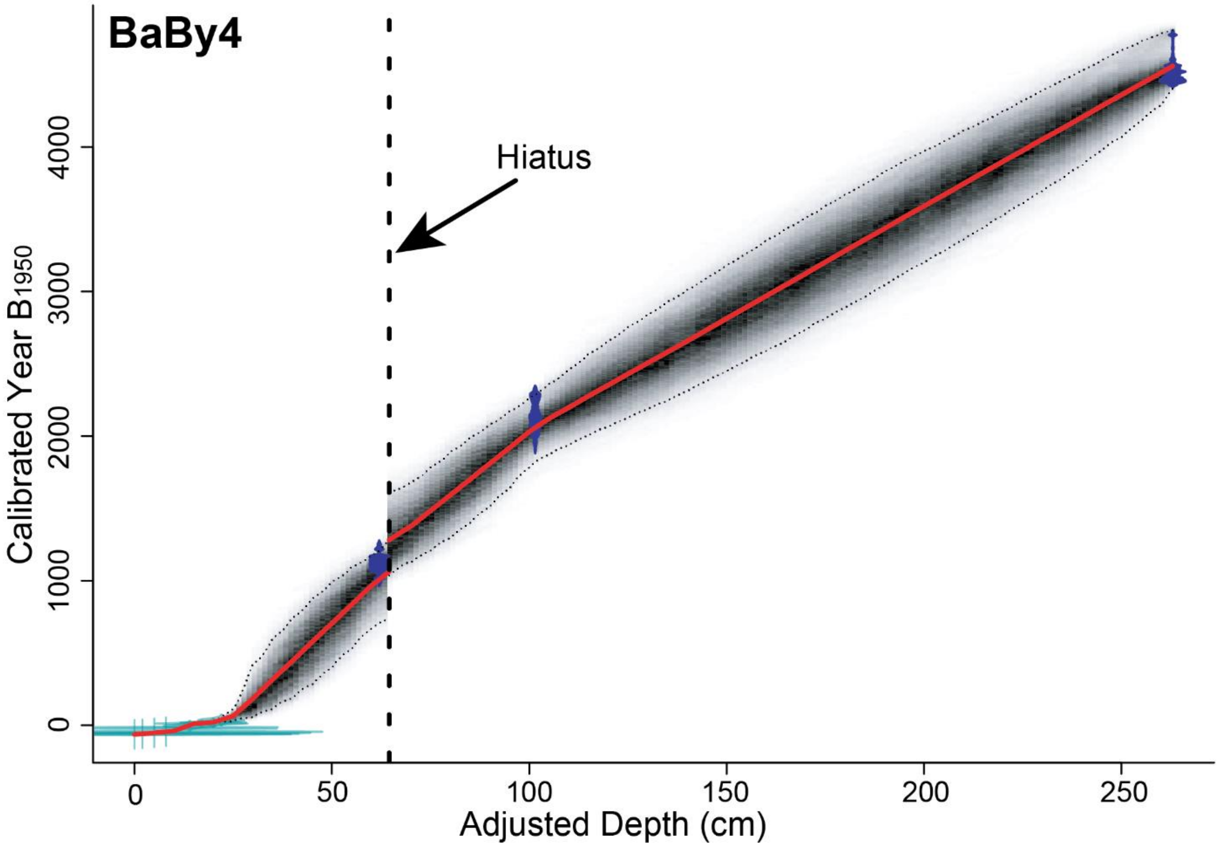

Supplementary Figure S1: Age model outputs from the Bacon age modeling package [43] for the BaBy4 sediment core. The adjusted depth is the depth after sand bed removal. Darker gray shades indicate a higher density of age-depth profiles. The 95% confidence interval is indicted by the black dotted lines bracketing the age-depth shaded curves, the weighted mean age-depth profile is indicated by the solid red line, calibrated age probabilities derived from radiocarbon ages are shown in blue, and ages derived from ^{210}Pb and ^{137}Cs are shown in green. A hiatus at 64.5 cm adjusted depth (102.5 cm composite depth with sand beds included) is indicated by a dashed vertical line.

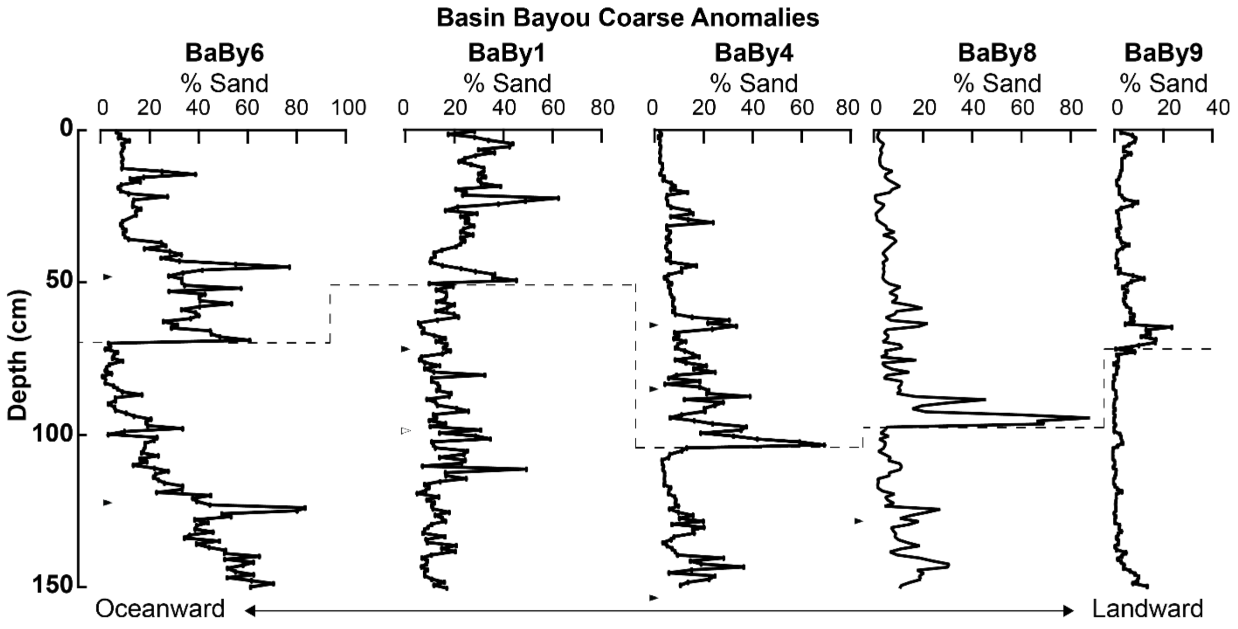

Supplementary Figure S2: Basin Bayou sediment sand content versus depth for the upper 150 cm in each core, which is the uppermost lithologic unit from which we derived the tropical cyclone reconstruction. The cores are listed in order of increasing distance from the baymouth barrier from left to right. The dashed line marks the erosional contact in each sediment core. Closed and open triangles mark the depths of radiocarbon dates measured on plant macrofossils and bivalves, respectively, from the upper 150 cm of each core. Radiocarbon ages and metadata for these dates are listed in Table 1 and Supplementary Table S4.

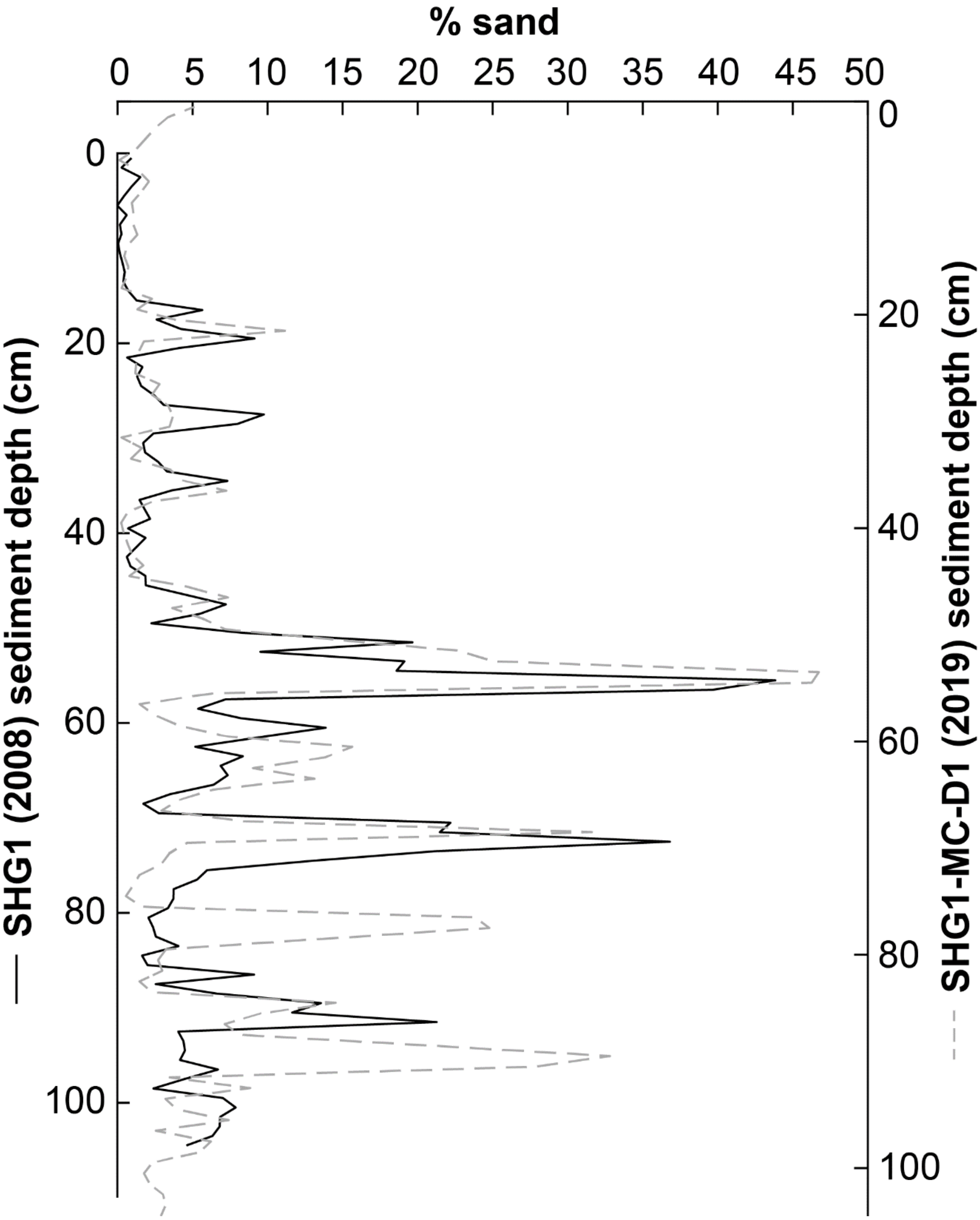

Supplementary Figure S3: Sand content for Shotgun Pond cores SHG1 (black solid line), collected in 2008, and SHG1-MC-D1 (gray dashed line), collected in 2019, are plotted versus core depth. A ~5% increase in sand content at the top of SHG1-MC-D1 likely represents Category 5 Hurricane Michael (2018). Based on nearby tide gauge data, the only tropical cyclone between the 2008 and 2019 core collection dates to exceed the minimum overwash regime storm tide on Bald Point was Hurricane Michael in 2018 (~2.3 m).

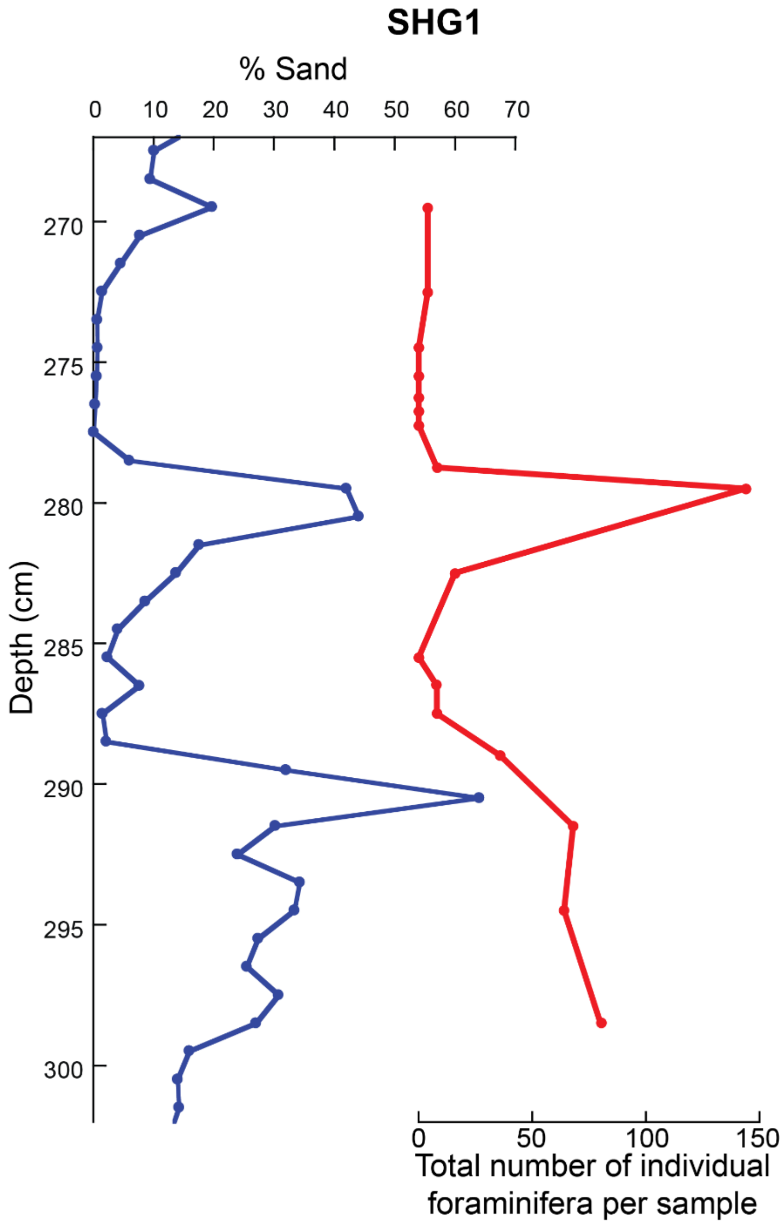

Supplementary Figure S4: Comparison of sand abundance and the total number of foraminifera along a 35-cm length of SHG1 (267-302 cm cumulative depth; adapted from ref. [15]).

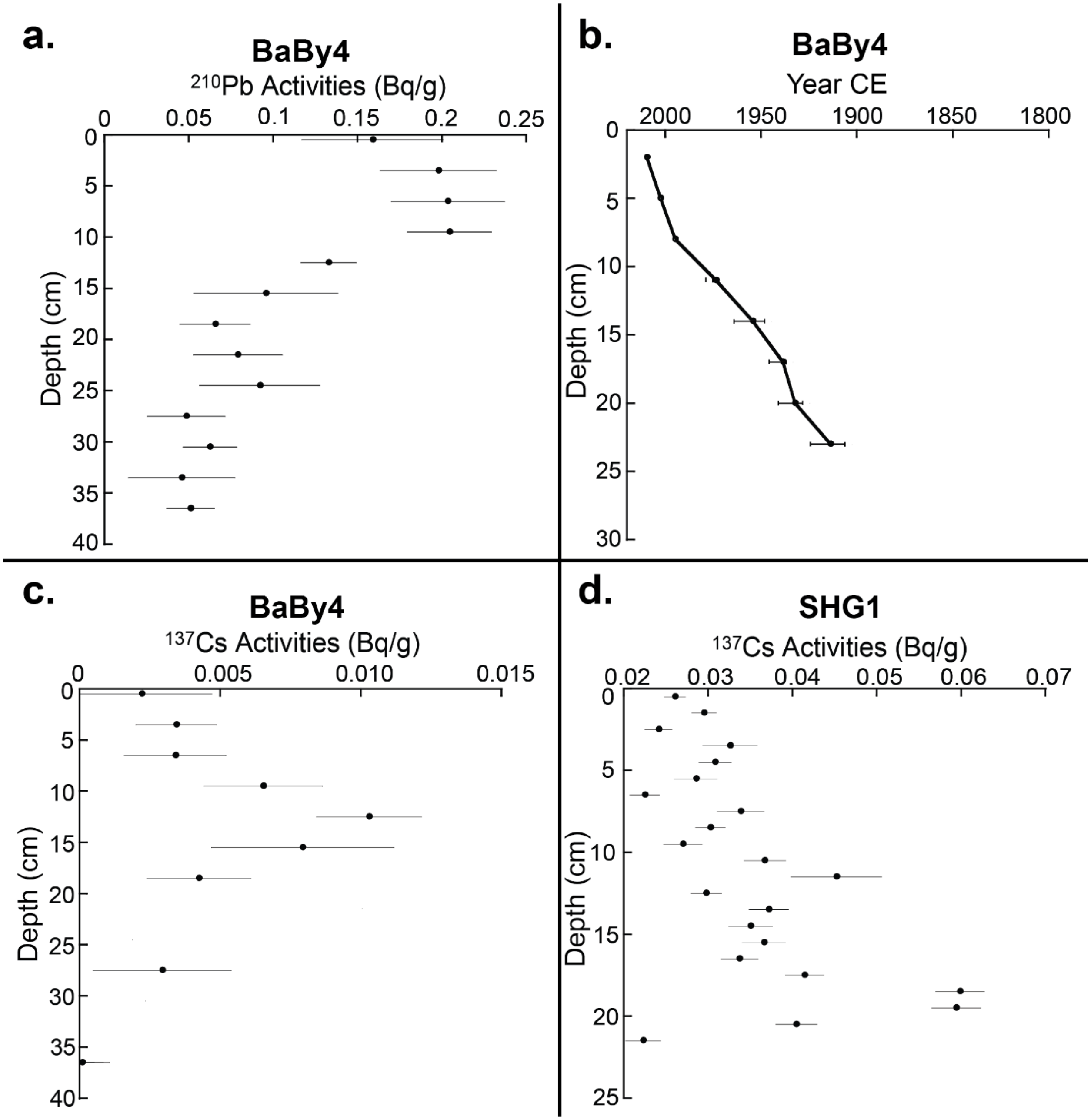

Supplementary Figure S5: ^{210}Pb and ^{137}Cs activities. a: ^{210}Pb activities vs. depth for BaBy4. b: ^{210}Pb ages vs. depth for BaBy4. c: ^{137}Cs vs. depth profile for BaBy4. d: ^{137}Cs vs. depth for SHG1. Horizontal lines in each panel indicate the analytical uncertainty range for each measurement.
